# A unifying gene signature for adenoid cystic cancer identifies parallel MYB-dependent and MYB-independent therapeutic targets

**DOI:** 10.18632/oncotarget.2985

**Published:** 2014-12-10

**Authors:** Ruli Gao, Chunxia Cao, Min Zhang, Maria-Cecilia Lopez, Yuanqing Yan, Zirong Chen, Yoshitsugu Mitani, Li Zhang, Maria Zajac-Kaye, Bin Liu, Lizi Wu, Rolf Renne, Henry V. Baker, Adel El-Naggar, Frederic J. Kaye

**Affiliations:** ^1^ Department of Medicine, Division of Hematology and Oncology, College of Medicine, University of Florida, Gainesville, FL, USA; ^2^ Genetics & Genomics Graduate Program, Genetics Institute, College of Medicine, University of Florida, Gainesville, FL, USA; ^3^ Department of Molecular Genetics and Microbiology, College of Medicine, University of Florida, Gainesville, FL, USA; ^4^ Department of Pathology, The University of Texas MD Anderson Cancer Center, Houston, TX, USA; ^5^ Department of Computational Biology and Bioinformatics, The University of Texas MD Anderson Cancer Center, Houston, TX, USA; ^6^ Department of Anatomy & Cell Biology, College of Medicine, University of Florida, Gainesville, FL, USA; ^7^ Department of Molecular Genetics, The University of Texas MD Anderson Cancer Center, Houston, TX, USA

**Keywords:** MYB salivary gland cancer, adenoid cystic cancer, extracellular matrix

## Abstract

MYB activation is proposed to underlie development of adenoid cystic cancer (ACC), an aggressive salivary gland tumor with no effective systemic treatments. To discover druggable targets for ACC, we performed global mRNA/miRNA analyses of 12 ACC with matched normal tissues, and compared these data with 14 mucoepidermoid carcinomas (MEC) and 11 salivary adenocarcinomas (ADC). We detected a unique ACC gene signature of 1160 mRNAs and 22 miRNAs. MYB was the top-scoring gene (18-fold induction), however we observed the same signature in ACC without detectable MYB gene rearrangements. We also found 4 ACC tumors (1 among our 12 cases and 3 from public databases) with negligible MYB expression that retained the same ACC mRNA signature including over-expression of extracellular matrix (ECM) genes. Integration of this signature with somatic mutational analyses suggests that NOTCH1 and RUNX1 participate with MYB to activate ECM elements including the VCAN/HAPLN1 complex. We observed that forced MYB-NFIB expression in human salivary gland cells alters cell morphology and cell adhesion in vitro and depletion of VCAN blocked tumor cell growth of a short-term ACC tumor culture. In summary, we identified a unique ACC signature with parallel MYB-dependent and independent biomarkers and identified VCAN/HAPLN1 complexes as a potential target.

## INTRODUCTION

Malignant epithelial salivary gland tumors (SGTs) represent a heterogeneous group of tumors, including the major subtypes of adenoid cystic carcinomas (ACC), mucoepidermoid carcinomas (MEC), and adenocarcinomas (ADC) [1]. Each of these malignant SGTs has no effective systemic treatment for patients who present or recur with unresectable disease and, until recently, there was little insight into the molecular basis for malignant salivary gland tumorigenesis. In 2003, the CRTC1-MAML2 fusion oncogene was isolated from a recurrent t(11;19) translocation in MEC [2]. This led to the discovery of a new CREB co-activator gene CRTC1 that is regulated by the Peutz-Jegher LKB1 kinase proposing a direct link between anabolic metabolism and salivary gland tumorigenesis [3-6]. MEC research efforts were facilitated by the availability of human tumor cell lines that allowed positional mapping of chromosomal breakpoints to isolate the etiologic fusion oncogene. In contrast, molecular characterization of a recurrent t(6;9) rearrangement in ACC was delayed due to lack of validated ACC tumor cell lines [7-10] until 2009 when the MYB-NFIB fusion oncogene was isolated using short-term cultures of ACC surgical biopsies [11]. ACC is a particularly aggressive subtype of SGT that frequently recurs with incurable metastatic disease many years after the initial surgical resection. Therefore, the identification of a recurrent MYB fusion event in ACC tumorigenesis provides an important clue to pursue new therapeutic strategies. MYB is a nuclear transcription factor that plays an essential role in the development and homeostasis of hematopoiesis [12-15]. In addition, MYB has been suggested to play a role in the development and homeostasis of selected glandular tissues including colon [16, 17] and breast [18]. In human cancer, tandem duplications of the *MYB* gene were detected in a subset of human T cell acute lymphoblastic leukemia (T-ALL) [19] and over-expression of MYB is associated with leukemia, breast and colorectal cancers [20-24]. Ectopic expression of MYB, however, has shown limited *in vitro* transforming activity that is largely restricted to hematopoietic animal model systems. Therefore, the identification of a recurrent MYB:NFIB translocation in ACC offered a new opportunity to study MYB biology in a defined epithelial human cancer model system.

The terminal NFIB exon, containing a small conserved open reading frame, is a recurrent translocation 3′ gene fusion partner in salivary gland ACC [11], pleomorphic adenomas (PA) [25] and lipoma [26]. Gründer et al.[25] indicated that this sequence resembled the CTD (C-terminal domain) of the largest RNA polymerase II subunit that can generate a physical bridge between the RNA transcription and pre-mRNA processing complexes through CTD-tail protein-protein interactions [27]. The functional role of NFIB in human cancer is unclear. For example, activation of HMGA2 by a reciprocal translocation with NFIB was proposed to arise by deletion of regulatory HMGA2 miRNA let-7 binding sites within 3′ untranslated (UT) sequences [28]. However, the detection of variant HMGA2 translocation breakpoints downstream of these consensus let-7 sites did not support this hypothesis [29]. MYB is also known to undergo 3′UT regulation in hematopoietic cell lineages and disruption of miRNA binding by the chromosomal translocation in ACC was recently proposed as a critical event for MYB activation [11]. Therefore, it is important to study both the functional properties of the novel MYB:NFIB fusion product as well as the biology and gene expression patterns in ACC samples that are both MYB fusion-positive and negative.

While MYB rearrangements were detected by FISH in approximately 50% of cases, there was an overall low rate of somatic mutations suggesting candidate cooperating driver mutations [30-32]. These data confirmed the importance of MYB activation but raised questions regarding downstream MYB signaling events in ACC tumorigenesis that might identify new therapeutic targets for patients with advanced disease.

To address these uncertainties, we have now performed global mRNA and miRNA expression analysis for a large collection of well-annotated malignant SGT and their matched normal tissues and integrated this data with somatic mutational analyses. We also performed functional assays to determine the transforming activity of activated MYB by either 3′ truncation or by fusion with terminal NFIB exon(s). We have detected a distinct ACC mRNA signature that includes over-expression of a network of extracellular matrix (ECM) components that arise from both MYB-dependent and -independent signals. These data suggests a cooperating role between MYB and RUNX1 /NOTCH1 signaling and proposes new therapeutic targets for this lethal disease.

## RESULTS

### ACC mRNA signature is independent of MYB rearrangement and enriched for cell membrane and extracellular matrix targets

**Figure 1:**
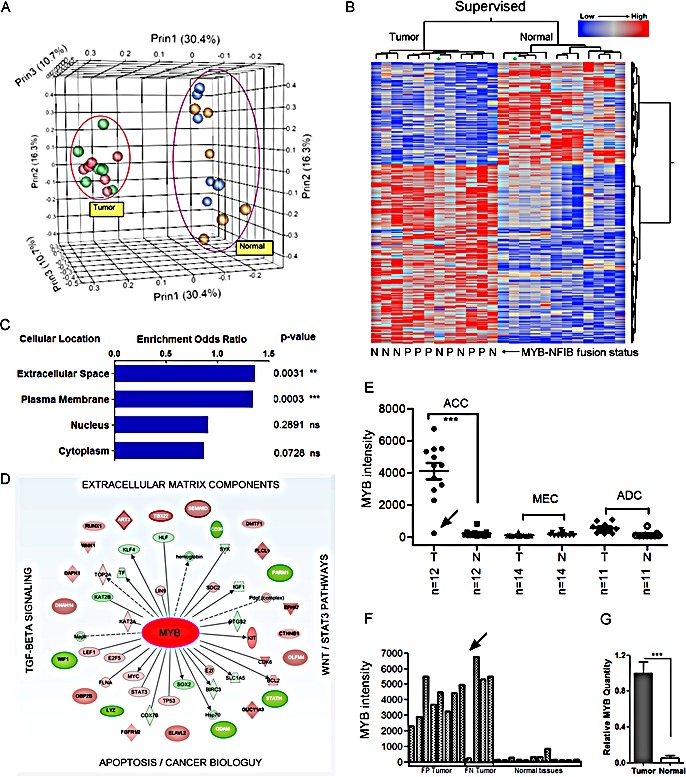
A unique mRNA gene signature distinguishes salivary gland adenoid cystic cancer (ACC) from matched normal salivary gland tissue A) Principle component analysis (PCA) for all salivary gland adenoid cystic cancer (ACC) and matched normal samples. Red and green spheres indicate fusion positive and negative tumors respectively. Blue and brown indicate their matched normal tissues respectively. B) Supervised hierarchical-ward clustering analysis using 1160 mRNA probe sets that were significantly expressed in ACC (two sides paired t-test, FDR=0.05; see Supplementary Table 1 for complete gene list). Star depicts tumor sample with negligible MYB expression and matched normal tissue. MYB-NFIB fusion positive samples (P) and fusion negative samples (N). C) Gene localization and molecular enrichment analysis using Fisher's t-test (***, paired t-test, two side p-value<0.0001; **, paired t-test, two side p-value<0.001; ns, paired t-test, two side p-value>=0.05). Gene localization was defined by Ingenuity IPA. D) MYB-related network in ACC. The inner layer: MYB-interaction genes defined by Ingenuity IPA. The outer layer, MYB regulating genes defined by published ChIP-Seq data [33]. E) Average intensities of MYB probe sets for three subtypes of salivary gland tumors and their matched normal samples (***, paired t-test, two side p-value<0.0001). F) MYB average intensity in ACC for each sample. Arrow depicts sample with low MYB. G) qRT-PCR validation of relative MYB expression levels in ACC tumors (***, two sample t-test, two side p-value<0.0001; error bars are S.E.M. of 12 samples).

To define a diagnostic adenoid cystic cancer (ACC) gene signature and to identify new therapeutic targets, we performed global mRNA and miRNA expression array analysis for 12 ACC tumors and matched adjacent normal samples. We also compared the ACC signatures to 14 mucoepidermoid cancers (MEC) and 11 salivary adenocarcinomas (ADC). All samples showed high RNA integrity (RIN >8) and passed quality controls described in Methods. We first performed principle component analysis (Figure 1A), both unsupervised (Supplementary Figure 1) and supervised (Figure 1B) clustering analysis for tumor and matched normal tissues to detect a unique mRNA signature that easily separates ACC from normal tissues. We detected 1160 genes that were differentially expressed in ACC as compared to matched normal tissues by using paired t-tests (Figure 1B, FDR=0.05, see Supplementary Table 1 for complete gene list). In addition, we were able to detect a secondary cluster across normal tissues that suggested a minor signature contribution from the specific tissue of origin (major vs minor salivary glands, Supplementary Figure 1). A striking finding was a distinct ACC mRNA signature regardless of tissue of origin that suggests a core mutational signaling pathway for this disease. This unique ACC mRNA signature was also markedly distinct from MEC and ADC mRNA signatures (data not shown). Although a recurrent MYB-NFIB fusion transcript is detected in many ACC samples, we and others have detected fusion-negative tumors in 20-40% of ACC samples. To test if MYB fusion-negative ACC tumors exhibit a different downstream gene expression profile, we initially pre-selected 6 fusion-positive and 6 fusion-negative ACC tumor samples scored by RT-PCR. As shown in Figure 1, there were no significant differences between these samples (Figure 1, panels A and B, Supplementary Figure 2, ANOVA FDR=0.05). We mined the 1160 genes with at least 2-fold change in ACC for predicted subcellular localization and functional properties using Fisher's t-test. We noted an enrichment of downstream gene targets that localized to the cell membrane, extracellular space, and extracellular matrix (Figure 1C). As expected, using the IPA analysis we also noted a marked enrichment in genes that were known MYB target genes or MYB interaction genes (Figure 1D). We further confirmed this observation by merging our dataset with the ChIP-Seq public MYB target genes list obtained using highly specific MYB antibodies [33], and showing that approximately 50% of the ACC signature were candidate MYB-related genes.

### Analysis of ACC tumors with negligible MYB expression identifies MYB-dependent and independent target genes

An unbiased inspection of global ACC gene expression identified MYB as the top differentially expressed gene as compared to either matched normal tissue or to other SGT subtypes such as MEC and ADC (Figure 1E, 1F, Supplementary Table 1). We confirmed this by independent qRT-PCR in the same set of ACC samples (Figure 1G). We also noted other previously identified ACC activation markers, such as c-KIT [34] and fibroblast growth factor receptor 1 (FGFR1) [35] that were up-regulated 5-fold and 4-fold respectively (Supplementary Table 1). The detection of MYB as the top differentially activated gene validates the accuracy of our data collection and methodology. However, we also noted a unique ACC sample with negligible MYB expression indistinguishable from basal levels in normal tissue, which, remarkably, exhibited an identical ACC mRNA gene signature (see asterix in Figure 1B and arrow in Figures 1E, 1F).

**Figure 2:**
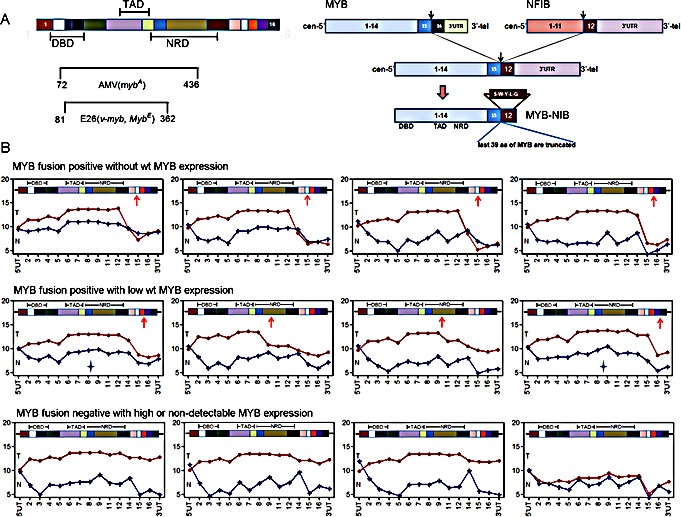
Exon array analysis is a sensitive tool to identify MYB C-terminal rearrangement status A) Illustrations of MYB protein structure and MYB-NFIB fusion events in ACC. DBD, DNA binding domain; TAD, transcriptional activation domain; NRD, negative regulation domain. B) MYB exon intensity plot for 12 ACC tumor samples identifies variable expression of MYB C-terminal exons and validates MYB fusion status. Red line, MYB exon intensities in ACC tumor; Blue line, MYB exon intensities in matched normal tissue; Star depicts two false fusion-positive samples validated by FISH.

The detection of a similar ACC mRNA gene signature in both MYB fusion-positive and fusion-negative tumors as well as a sample with low MYB expression suggested several alternative mechanisms: i) presence of subtle variant MYB fusion events that were not detected in our samples with RT-PCR. ii) alternative, non-fusion somatic events that activate MYB, or iii) activation of an alternative gene pathway that mimics activated MYB signaling. To address the first possibility, we re-analyzed the relative expression levels of MYB exons using detailed exon array expression data. This microarray platform allows for multiple probes covering most MYB exons and we are able to generate ‘exon-plot’ images to examine exon-specific expression except for MYB exon 10 that was not present in ACC tumors. As expected, we were able to infer the approximate MYB breakpoints by noting relative drops in average probe set intensities immediately after the breakpoints in validated MYB-NFIB fusion positive samples. In contrast, true fusion-negative samples showed consistent probe intensities across the whole gene. With this ‘exon-plot’ approach, we identified two misdiagnosed ‘fusion negative’ tumors (Figure 2B, asterix), which were then validated for MYB rearrangements by FISH (data not shown). After these corrections, however, we were still unable to detect significant differences in downstream mRNA gene signature between MYB fusion-positive and the remaining fusion-negative tumors (Supplementary Figure 2). It is possible that cryptic MYB 3′ rearrangements may have escaped detection of the exon array probes and FISH analyses or, alternatively, these data may suggest that MYB activation is not necessarily dependent on 3′ structural rearrangement to drive ACC downstream signaling pathway. Inspection of the MYB 3′ terminal exon array data also allowed us to infer co-expression of the reciprocal and/or remaining wildtype (wt) MYB allele in fusion-positive samples. For example, we could infer the absent expression of both reciprocal and wildtype (wt) MYB alleles in at least 4 ACC samples (Figure 2B) suggesting a minor role for these alleles in ACC tumorigenesis.

**Figure 3:**
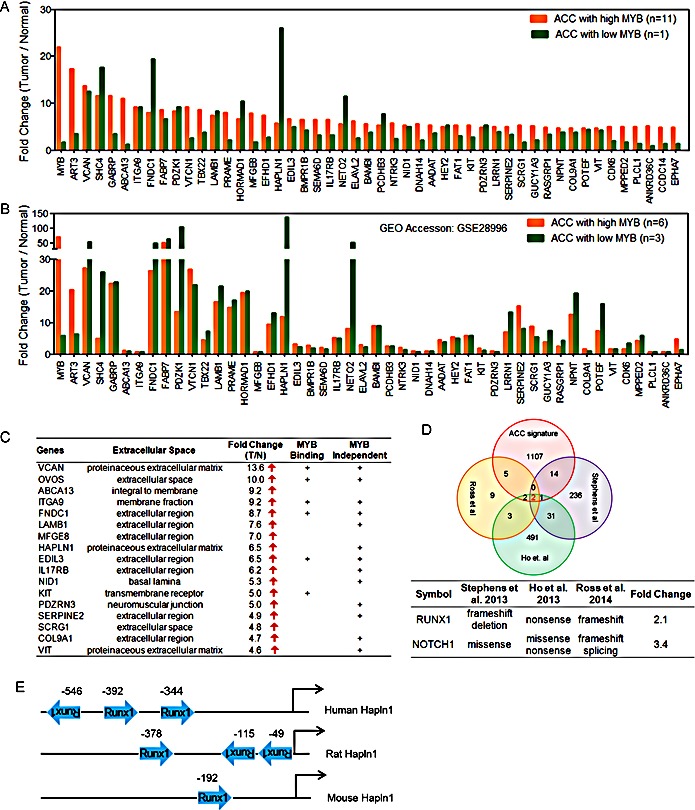
Integrative analysis identifies both MYB-dependent and MYB-independent genes in ACC A) Differentially expressed genes with the highest fold change compared to normal in high or low MYB ACC tumors indicate extracellular gene activation. B) The same analysis as panel A was performed on ACC gene expression array data (Affy_HG133) obtained from public GEO database (accession: GSE28996). C) Top scoring genes that are components of extracellular matrix (ECM). MYB binding genes were defined by published ChIP-Seq data. MYB independent targets were defined as genes that were activated in both high and low MYB ACC tumors. D) Integrative analysis of ACC gene signature to recently published ACC mutational sequencing data identifies potential roles of RUNX1 in ACC tumorigenesis. E) Regulation of extracellular gene HAPLN1 by RUNX1.

As noted in Figure 1, we detected a unique ACC tumor sample with negligible MYB expression that unexpectedly retained the diagnostic ACC mRNA signature. We pursued this observation as a tool to search for potential MYB-dependent and MYB independent targets genes in ACC tumorigenesis. To validate this observation, we first searched the public gene array database for additional ACC cases and detected 3 independent ACC tumors with negligible MYB expression (GSE28996 [10]). These MYB-low ACC tumor samples were tested at different times on different array platforms but still showed an identical ACC downstream gene expression patterns indicating both MYB-dependent and independent gene transcripts (Figure 3A-C) that were enriched for extracellular matrix products (Figure 3C).

**Figure 4:**
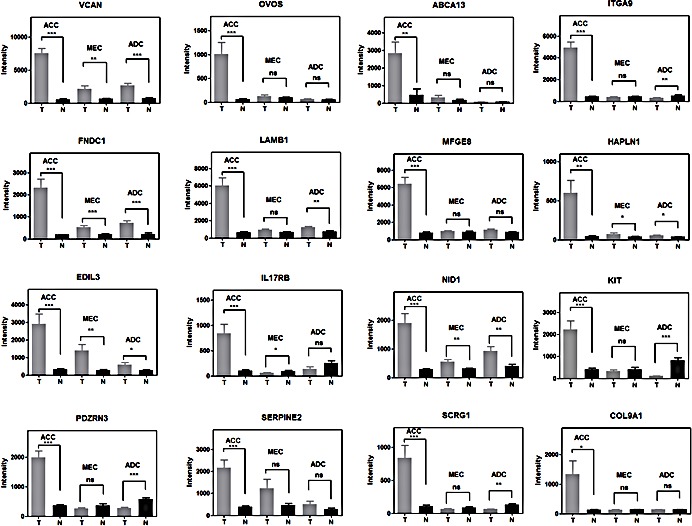
Comparison of the differential ECM activation in three subtypes of salivary gland tumors and their matched normal samples ***, t-test, two side p-value<0.0001; **, p-value<0.001; *, p-value<0.05; ns, p-value >=0.05. Error bars are S.E.M. of 12 replicates of ACC, 14 replicates of MEC and 11 replicates of ADC.

We then integrated our new ACC gene signature with data from three recent whole exome ACC sequencing studies [30-32] and identified RUNX1 and NOTCH1 as the only overlapping genes targeted for potential gain of function mutations (missense or C-terminal truncated protein) and associated with elevated gene expression (Figure 3D). It is also notable that the clustering of MYB, NOTCH1, RUNX1, and FBXW7 mutations seen in this uncommon epithelial tumor ACC overlaps with frequent somatic mutations seen in human acute lymphocytic leukemia patients [36]. Approximately 30% of the top-scoring gene loci in ACC encode components of the extracellular matrix including the HAPLN1/VCAN binding partners (Figure 3A-C). We also noted that RUNX1 (aka AML1/CBFA/PEBP2) cooperates with MYB to activate enhancer elements for many eukaryotic genes [37-40] and has conserved binding sites upstream of the human HAPLN1 promoter (Figure 3E) necessary for *HAPLN1* expression [41, 42]. These data suggest that activated RUNX1 could participate as a co-activator in ACC for the VCAN/HAPLN1 complex. Although these extracellular components can be upregulated in other tumor lineages, we noted that the top 16 differentially expressed ECM genes on the basis of fold-induction were specifically observed in ACC but not in the other two SGT subtypes of MEC and ADC (Figure 4).

### Global miRNA signature distinguishes ACC from normal salivary gland tissues

**Figure 5:**
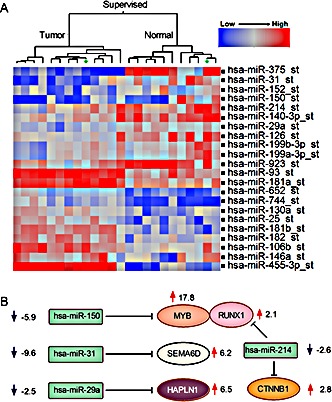
Distinct miRNA expression in ACC tumorigenesis A) Supervised hierarchical ward clustering for ACC using 22 mature miRNA seed probe sets that are significantly expressed (two sides paired t-test, FDR=0.05). B) Regulatory miRNA target predictions (TargetScan, Pictar) suggest potential roles of miRNAs in regulation of extracellular genes in ACC tumorigenesis.

Since MYB expression can be regulated by miRNA binding, deletion of these 3′ UT binding sites were proposed as a primary mechanism for MYB activation in ACC tumors. Therefore, to test this hypothesis, we performed global unbiased miRNA microarray analyses for the same collection of ACC and adjacent normal tissue samples. We tested a total of 847 probes with each probe representing a distinct human mature miRNA species. We detected a unique ACC miRNA profile distinct from matched normal samples (Figure 5A, see Supplementary Table 2 for complete list) and distinct from MEC and ADC tumors (data not shown). However, we again noted no significant difference in the miRNA signature between MYB fusion positive samples with deleted 3′UT sequences and fusion-negative ACC samples that are predicted to retain these miRNA binding sites. These data do not exclude a role for miRNA regulation of MYB since the MYB inhibitory *hsa-miR-150* was down-regulated in all ACC tumors (Figure 5A). However, they argue against miRNA binding sites as a specific target for the recurrent t(6;9) chromosomal translocation. We also integrated the miRNA data with our global mRNA gene expression data and noted potential regulatory interconnections including patterns of ECM components gene expression (Figure 5B). For example, the HAPLN1 inhibitory hsa-miR-29 was down-regulated 2.5 fold (Figure 5B).

### Forced expression of MYB-NFIB alters human salivary gland cell morphology and adhesion *in vitro*

**Figure 6:**
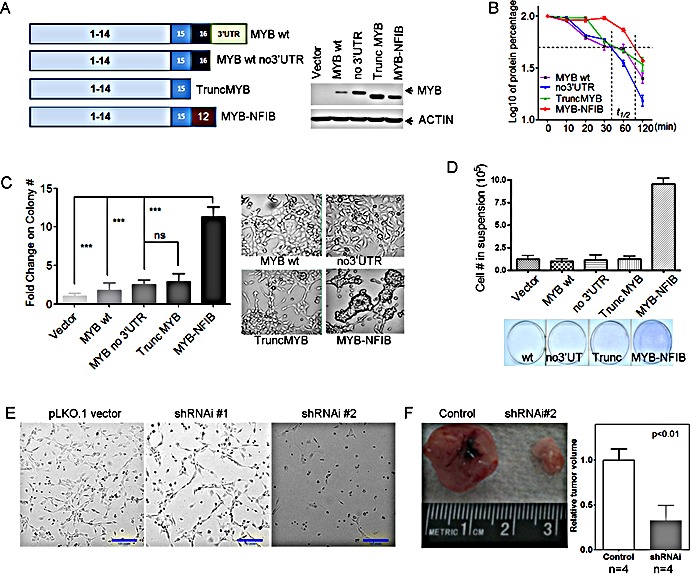
Functional analysis of MYB fusion protein A) Illustration of MYB cDNA clones tested fragments tested and their expression efficiency in human immortalized salivary ductal cells (DC). MYB-NFIB protein showed increased steady-state protein and B) prolonged half-life in human immortalized myoepithelial cells (MC). C) Forced expression of MYB-NFIB enhanced the anchorage independent colony formation of human salivary DC cells. ***, t-test, two side p-value<0.0001; ns, p-value >=0.05. Error bars are S.E.M. of 3 replicates for each group. D) Decreased attachment and increased viability of suspension cells after ectopic MYB-NFIB expression. Error bars are S.E.M. of 3 replicates for each group. E) ACC tumor cell growth inhibition after shRNAi depletion of VCAN, and F) decreased xenograft tumor growth ability in NOD.SCID mice (two sample t-test, error bars are S.E.M. for 4 replicates).

To study functional roles of MYB and MYB-NFIB in ACC tumorigenesis, we generated expression vectors including full-length MYB with intact 5′ and 3′ UT (MYB wt); MYB with only 5′UT (MYB no 3′UTR), truncated MYB with a stop codon inserted at the most common translocation breakpoint in exon 15 (Trunc MYB), and MYB-NFIB fusion (MYB-NFIB) (Figure 6A). We obtained normal human salivary gland cells that were immortalized with SV40 to select clones that morphologically represent ductal cells (DC) [43] for transient and stable transfection with the MYB constructs. We observed lower MYB protein levels after either transient or stable expression of cDNA constructs that retained 3′ UT regions (Figure 6A). We also detected a modest, but statistically significant prolongation of MYB protein half life in the MYB-NFIB fusion product as compared to the truncated or wt MYB protein (Figure 6B). Interestingly, we detected clusters of loosely attached oncospheres as well as enhanced colony growth in soft agar of the DC cells transfected with the fusion MYB-NFIB product as compared to other MYB constructs (Figure 6C). We also noted that these cells were more loosely attached to the plastic dishes *in vitro* which we measured by counting viable cells in the supernatant at day 4 after initial plating and then reseeding these cells onto fresh dishes to monitor for growth by crystal violet staining (Figure 6D). A similar morphological appearance was detected when MYB-NFIB was transfected into primary mouse salivary gland cells (Supplementary Figure 3). These data, as well as the ACC mRNA gene expression signature identified in Figure 1, suggests cell membrane and extracellular candidate genes as a focus for new therapeutic targets in ACC. We selected VCAN from our data since this target was the highest expressed ECM gene target in our expression studies and VCAN (aka CSPG2) belongs to a family of versican proteoglycan genes with broad cellular functions including cell morphogenesis, adhesion, migration, angiogenesis, inflammation and growth factor regulation. VCAN was also previously shown to suppress tumor cell growth in mesothelioma, osteosarcoma, prostate, and other tumor cell lineages suggesting it may be a promising therapeutic target for preclinical development [44]. Since there are no validated ACC tumor cell lines [7-10] we selected a short term culture of a human ACC biopsy collected through an IRB approved protocol and treated cells with either control or 2 different lentiviral VCAN shRNAs. We detected marked growth inhibition of ACC tumor cells *in vitro* (Figure 6E) and also reduced nude mouse xenograft tumor growth *in vivo* (Figure 6F) with both VCAN shRNAs.

## DISCUSSION

ACC is one of the most common malignant SGT subtypes with no curative strategy for unresectable disease. In addition, lethal metastatic recurrences can present many years after the initial surgical resection that has further stimulated an urgent search for new therapies. Cytotoxic chemotherapy has not been effective for advanced disease and clinical trials testing imatinib to target KIT over-expression [45] or lapatinib for HER activation [46] have been unsuccessful for ACC; Although activation of FGFR has been proposed as another candidate target for ACC that is currently under clinical investigation; more work is needed to define the core cancer signaling pathways and to identify new cancer treatment targets. A critical clue towards this goal was the detection of a recurrent MYB-NFIB fusion transcript in a subset of ACC tumors, however there are numerous obstacles preventing the direct targeting of a nuclear transcription factors, such as MYB, with pharmacological or biological agents. To address this, we have now pursued a global nonbiased search for new ACC-specific mRNA and miRNA signatures that would identify candidates genes localized on the cell membrane or extracellular space that would be more accessible for therapeutic interventions.

In fact, we discovered that 30% of the top ranking genes measured by fold overexpression were localized within the extracellular space and this pattern appeared unique for ACC as compared to other SGT subtypes such as mucoepidermoid carcinomas and adenocarcinomas. Unexpectedly, we also detected an ACC sample with low MYB expression that still retained this global ACC gene expression signature. We validated this observation by identifying 3 additional ACC tumor samples from the public microarray databases with low MYB levels suggesting that approximately 5-10% of ACC samples may have this alternate phenotype with negligible MYB levels. Although MYB activation defines ACC tumorigenesis and MYB overexpression has been associated with a worse clinical outcome in patients with ACC [11, 47-49], there may be heterogeneity of MYB staining within tumor samples. For example, Costa et al (2014) showed that highly transformed area of ACC tends to have lower MYB expression although not statistically significant (p=0.123, Wilcoxon test), and they also observed an absence of MYB expression in 1 case (out of 8 ACC cases) [50]. We hypothesize that ACC tumors with negligible MYB expression may have acquired an alternate somatic alteration in a gene target that mimics activated MYB signaling.

To pursue this possibility we performed an integrated analysis of gene expression and somatic mutational data and these data proposed a role for MYB, NOTCH1, and RUNX1 in regulating a subset of key ACC target genes. RUNX1, a MYB transcriptional co-factor, may be a specific target for missense or C-terminal truncated activating mutations in ACC that cooperate to deregulate MYB transcriptional activation [51-55]. For example, MYB is required for both RUNX1 transcriptional activity [54, 56], for the ability of a chimeric RUNX1 fusion oncogene to transform hematopoietic cells [54, 57, 58], and RUNX1 can cooperates with MYB to activate ectopic targets through novel MYB binding sites in cancers [53]. Interestingly, we discovered that two of the most highly expressed ACC target genes, VCAN and HAPLN1, can bind within a predicted ECM complex in ACC tumor cells and, in the case of HAPLN1, appear to be regulated by RUNX1 activation [41, 42]. In addition to the ability of RUNX/NOTCH to cooperate with MYB for promoter activity [59], others have noted that either activated NOTCH or aberrant accumulation of VCAN can induce the same heart valve phenotype in rodent models suggesting another connection between NOTCH/RUNX/MYB and ECM regulations [60]. VCAN/HAPLN1 binding in the ECM is predicted to activate a broad range of signaling pathways including the epidermal growth factor receptor (EGFR), TGF-β, insulin-like growth factor receptor (IGFIR) and others [61, 62]. The pro-tumorigenic role of VCAN/HAPLN1 complex has been tested in several other tumor types, such as malignant pleural mesothelioma [63] and breast and prostate cancers and increased tumor-specific VCAN expression levels have been reported in ACC and several other tumor types [64-69]. For example, VCAN was found to be 29-fold up-regulation in human ACC tumors comparing to normal tissues [69] and increased VCAN protein staining was detected in mouse xenograft tumor models for human ACCs [10]. Although stromal VCAN expression was associated with a poor prognosis in oral squamous cell carcinoma [70] and was associated with high rate of tumor recurrence and more advanced disease in non-small cell lung cancers [71], our data does not yet confirm a direct link between VCAN overexpression and many of the clinicopathological and prognostic features in ACC. We have now demonstrated tumor growth inhibition *in vitro* and *in vivo* with short term ACC cell cultures following depletion of VCAN and the generation of new validated immortalized ACC tumor cell lines and animal models will allow preclinical testing of these new promising extracellular targets for development of new treatment strategies.

## METHODS

### RNA extraction, quality control and quantification

A total of 74 salivary gland tumors including 24 salivary gland adenoid cystic cancer, 28 mucoepiderrmoid cancer and 22 adenocarcinoma samples were excised from human patients under approved IRB protocols from University of Texas MD Anderson Cancer Center. MYB-NFIB fusion positive tumors were preselected by RT-PCR. Total RNAs were extracted and evaluated using NanodropTM 8000 (ThermoFisher Scientific, MA) and the Agilent 2100 Bioanalyzer with RNA 6000 Nano Labchip® (Agilent Technologies, CA).

### Microarray assay and data analysis

Microarray assays were performed by following manufacturer's instructions (GeneChip ^®^_human gene 1.0_ST and miRNA_GeneChip_1.0; Affymetrix, CA). Data were processed through the robust multichip average algorithm and quantile normalization methods followed by log2 transformation [72, 73]. Processed data were analyzed using SAS JMP^®^Genomics (SAS Institute, NC). Both baseline filter (intensity >=50) and variation filter (|fold-change|>=2) were applied before mRNA differential expression analysis. Both baseline filter (intensity >=800) and variation filter (|fold-change|>=2) were applied for microRNA chip analysis. Hierarchical cluster analysis and the principle component analysis were performed for all samples using SAS (SAS Institute, NC). Paired t-tests were performed to compare tumor samples to their matched normal tissues and Hochberg-Benjamini FDR was set at 0.05 [74]. The mixed ANOVA analysis was performed for the comparison between MYB-NFIB fusion-positive and fusion-negative tumors. All data were deposited in the NCBI GEO database (GSE59702). MYB interaction genes were extracted from IPA interactions (Ingenuity IPA, Redwood City, CA). MYB target gene was downloaded from public ChIP-Seq data that listed all genes having significant MYB binding sites in their promoters [33]. Gene enrichment analysis was performed using Fisher's test.

### Plasmid construction, transfection, cell culture and shRNAi knock-down

*MYB* fragments were amplified from commercial *MYB* cDNA clone (Open Biosystems, AL, Cat MHS1010-9206139) and inserted into the pAcGFP1N1 expression plasmid using NotI and KpnI restriction sites and the terminal NFIB exon with or without NFIB 3′UT sequences was introduced by PCR-mediated cloning. All constructs were validated by sequencing. For stable clone selection, 50 ng/ml Geneticin^®^ selective antibiotics (Gibco^®^, Cat #11811-098) was introduced after 48 hours of transfection. The pLKO.1 lentiviral VCAN-shRNA constructs were obtained from Open Biosystems (Huntsville, AL). The lentiviral procedures applied for shRNAi were performed as described in [75]. The pBABE-puro-hTERT plasmid was purchased from Addgene (MA, plasmid1771).

NIH/3T3 cells were cultured in DMEM (Sigma) supplemented with 10% FBS and 1% pen/strep (Gibco^®^). HEK293T cells were cultured in high glucose DMEM medium supplemented with 10% FBS and 1% pen/strep (Gibco^®^). RK3E cells were cultured in RPMI 1640 (Sigma) with 10% FBS and 1% pen/strep (Gibco^®^). Normal human immortalized salivary gland cells [76] were generous gift from Dr Stephen Hsu at Medical College of Georgia and cells were cultured with the Keratinocyte-SFM medium with L-Glutamine, EGF and BPE (Gibco^®^, Cat17005-075). *Defined Trypsin Inhibitor* was also purchased from Invitrogen (CatR-007). The primary normal salivary gland cell culture for wt FVB mice (The Jackson Laboratory, Jacksonville, FL) was performed following procedures described in [76]. Cells were observed under Leica microscope (Leica microsystems, IL).

### Quantitative RT-PCR

Reverse transcription and amplification of cDNA was performed in the 7900 HT Fast (Applied Biosystems) system by following manufacture instruction using High-Capacity cDNA Reverse Transcription Kits (Applied Biosystems^®^, Cat4368814) and TaqMan Fast Universal PCR Master Mix (Applied Biosystems^®^, Cat4367846). All gene expression probes were commercially purchased (Applied Biosystems^®^). Relative expression of target gene was calculated in comparison to 18s rRNA values. The quantitative miRNA RT-PCR analysis was performed by following procedures as described in [77].

### Western-blot, and antibodies

Cells pellet was lysed in RIPA buffer (Boston Bioproducts) and subjected to SDS-polyacrylamide tris-glycine gel separation (Invitrogen). And the membrane transfer was performed using iBlot® 7 Minute Blotting System (Invitrogen) following the manufacturer's instructions. Membrane was blocked with 3% BSA overnight at 4°C and then incubated with primary antibodies for 1 hour at room temperature. Antibody against N-terminus MYB was purchased from Abcam (EP769Y). After washed with PBS, the membrane was incubated with a horseradish peroxidase-conjugated secondary antibody for 45 minutes. Bands were visualized with Chemiluminescence (Pierce).

### Cycloheximide blocking assay

The protein half life was determined by cycloheximide blocking assay. Cells were seeded into 100mm dish and subjected to inhibition of protein synthesis with 100 μg/ml cycloheximide (Sigma) for 10 minutes, 20 minutes, 30 minutes, 60 minutes, 120 minutes or no treatment as control. Cycloheximide treated cells were then harvested and subjected to SDS-PAGE gel separation and western-blot analysis for retained protein. The relative protein quantity were measured using ImageJ [78].

### Soft-agar assay for anchorage independent colony formation

A total of 1×10^5^ trypsinized cells were subjected to soft-agar assay as described in [79]. Generally, top agar was prepared with 0.8% Difco Agar and 0.2% Difco peptone and then mixed with 2x Media (Gibco). Base agar was prepared with 1.6% Difco Agar and 0.4% Difco peptone and then mixed with 2x Media (Gibco). Triplicates were set for each cell construct. Agar plates were then put into incubator (Thermo Scientific) at 37°C and 5% CO_2_ for 3 weeks. And cells were feed once every week. Colonies were counted manually under10x microscope (Leica microsystems, IL).

### Self-suspension viable cell growth

A total of 1×10^6^ cells were seeded into 6-well plates in day one, and then viable cells in the culture supernatant were counted with TrypanBlue (Gibco) staining at day 4. Unstained detached cells were washed twice with PBS, reseeded to 6-well plates for growth and then stained with crystal violet for cell density observation after 4 days.

### Xenograft tumor model and primary cell culture

A total of 1×10^6^ cells were washed twice with PBS and then subjected to subcutaneous flank injection into NOD.SCID mice (The Jackson Laboratory, Jacksonville, FL). Freshly prepared human ACC tumor obtained under IRB-approved tissue protocol was also subjected to xenograft tumor growth and short term cell culture. Xenograft tumor volumes were calculated as: volume = (width)^2^ × length/2. The primary tumor cell culture was performed using methods as described in [43, 76]. The media used for primary tumor cell culture includes F12K (Sigma) 1:1 mixture with DMEM (Sigma) supplemented with 5-20% FBS (Gibco) and ACL4 medium supplemented with 5% FBS.

## SUPPLEMENTARY MATERIAL FIGURES AND TABLE


