# Supplementary material for: A unifying gene signature for adenoid cystic cancer identifies parallel MYB-dependent and MYB-independent therapeutic targets
Source: Oncotarget. 2014 Dec 10;5(24):12528–42. doi: 10.18632/oncotarget.2985 (PMC4350357; doi:10.18632/oncotarget.2985)
Supplement: Supplementary file 1 [file oncotarget-05-12528-s001.pdf]

A unifying gene signature for adenoid cystic cancer identifies parallel MYB-dependent and MYB-independent therapeutic targets

Supplementary Material

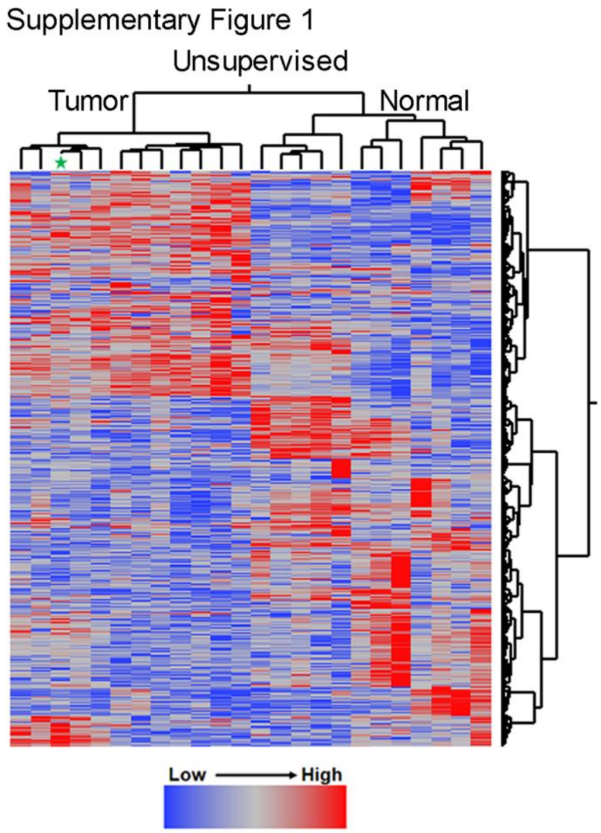

Supplementary Figure 1: ACC tumor has distinct gene expression profile from the matched normal tissues. Unsupervised hierarchical 2-way ward clustering analysis for ACC using 4027 annotated probe sets with Cov>0.3.

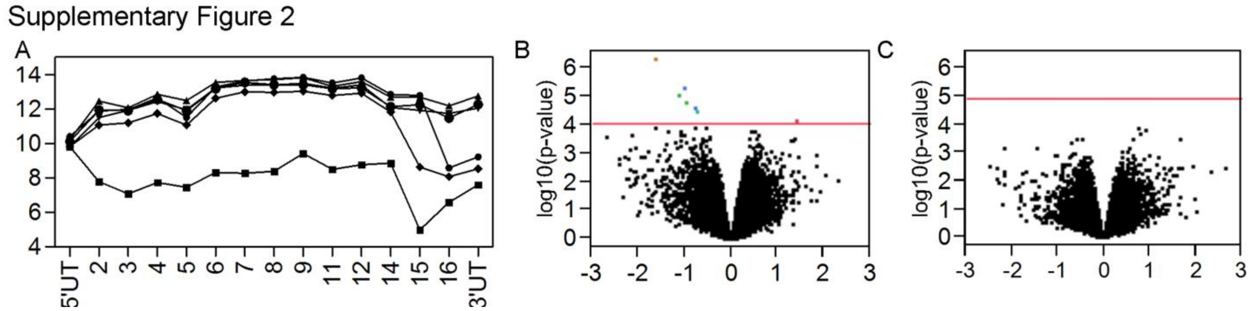

Supplementary Figure 2; Comparison between MYB-NFIB fusion positive and fusion negative ACCs. A) MYB exon plot for preselected fusion negative ACC tumors. One fusion negative tumor had low MYB expression and two 'fusion negative' tumors were identified as misclassified on the basis of loss expression of terminal MYB exons. B) and C) Volcano plot of significance levels against log2 gene difference between tumors and matched normal samples before and after fusion status correction respectively (FDR=0.05).

Supplementary Figure 3

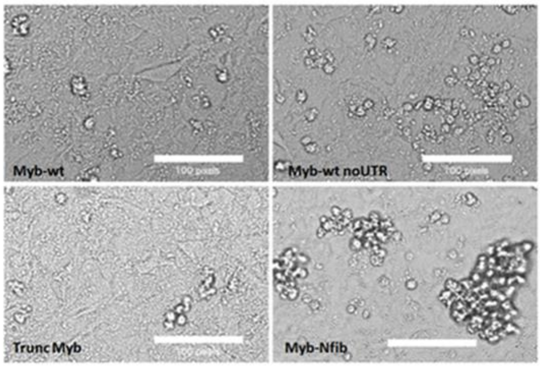

Supplementary Figure 3: Altered morphology with oncospheres after forced expression of MYB-NFIB changed in short-term cultured wt FVB mouse salivary gland cells. Murine salivary gland cells were co-transfected with hTERT and MYB-/NFIB expression plasmids. Pictures were taken under 10x microscopy.

Supplementary Table 1: Significantly expressed genes in adenoid cystic cancers compared to matched normal tissues. Paired t-test, FDR=0.05; Baseline filter: max{tumor, normal}>=50; Baseline filter: |fold change|>=2.0.

probeset_id	Symbol	Gene_Name	FC_T/N
8122202	MYB	v-myb myeloblastosis viral oncogene homolog (avian)	17.8
8095806	ART3	ADP-ribosyltransferase 3	15.2
8106743	VCAN	versican	13.6
7988563	SHC4	SHC (Src homology 2 domain containing) family, member 4	12.0
8109926	GABRP	gamma-aminobutyric acid (GABA) A receptor, pi	10.6
7953873	OVOS	ovostatin	10.0
7961026	LOC728715	ovostatin homolog 2-like	9.3
8132743	ABCA13	ATP-binding cassette, sub-family A (ABC1), member 13	9.2
8078619	ITGA9	integrin, alpha 9	9.2
8123104	FNDC1	fibronectin type III domain containing 1	8.7
8121784	FABP7	fatty acid binding protein 7, brain	8.5
7904843	PDZK1	PDZ domain containing 1	8.3
7918936	VTCN1	V-set domain containing T cell activation inhibitor 1	8.3
8168534	TBX22	T-box 22	8.2
8142194	LAMB1	laminin, beta 1	7.6
8117170	ENSG00000222515	ncrna:misc_RNA chromosome:GRCh37:6:22085773:22086147:1	7.3
8074856	PRAME	preferentially expressed antigen in melanoma	7.2
7919787	HORMAD1	HORMA domain containing 1	7.0
7991234	MFGE8	milk fat globule-EGF factor 8 protein	7.0
8074168	Broad TUCP	linc-OR4Q3-3 chr14:+:19650031-19684290	6.9
8049187	EFHD1	EF-hand domain family, member D1	6.8
8112971	HAPLN1	hyaluronan and proteoglycan link protein 1	6.5
8112980	EDIL3	EGF-like repeats and discoidin I-like domains 3	6.5
8096511	BMPR1B	bone morphogenetic protein receptor, type IB	6.3
7983527	SEMA6D	sema domain, transmembrane domain (TM), and cytoplasmic domain, (semaphorin) 6D	6.2
8080562	IL17RB	interleukin 17 receptor B	6.2
8050007	PXDN	peroxidasin homolog (Drosophila)	6.1
8001197	NETO2	neuropilin (NRP) and tolloid (TLL)-like 2	6.1
8160459	ELAVL2	ELAV (embryonic lethal, abnormal vision, Drosophila)-like 2 (Hu antigen B)	5.8
8055153	POTEE	POTE ankyrin domain family, member E	5.6
8144228	FLJ36840	uncharacterized LOC645524	5.5
7926875	BAMBI	BMP and activin membrane-bound inhibitor homolog (Xenopus laevis)	5.5
8140035	FLJ26938	Homo sapiens cDNA FLJ26938 fis, clone RCT07169.	5.5
8108688	PCDHB3	protocadherin beta 3	5.4
7991186	NTRK3	neurotrophic tyrosine kinase, receptor, type 3	5.4
7925320	NID1	nidogen 1	5.3
8054037	RN5S101	RNA, 5S ribosomal 101	5.2
7910047	DNAH14	dynein, axonemal, heavy chain 14	5.2
8164843	OBP2B	odorant binding protein 2B	5.1

probeset_id	Symbol	Gene_Name	FC_T/N
8122202	MYB	v-myb myeloblastosis viral oncogene homolog (avian)	17.8
8103706	AADAT	aminoadipate aminotransferase	5.1
8121850	HEY2	hairy	5.1
8104079	FAT1	FAT tumor suppressor homolog 1 (Drosophila)	5.1
8095110	KIT	v-kit Hardy-Zuckerman 4 feline sarcoma viral oncogene homolog	5.0
8088848	PDZRN3	PDZ domain containing ring finger 3	5.0
8077366	LRRN1	leucine rich repeat neuronal 1	5.0
8059376	SERPINE2	serpin peptidase inhibitor, clade E (nexin, plasminogen activator inhibitor type 1), member 2	4.9
8017039	SEPT4	septin 4	4.9
8103736	SCRG1	stimulator of chondrogenesis 1	4.8
8097957	GUCY1A3	guanylate cyclase 1, soluble, alpha 3	4.8
8027348	ZNF730	zinc finger protein 730	4.8
8013272	CCDC144A	coiled-coil domain containing 144A	4.8
7987405	RASGRP1	RAS guanyl releasing protein 1 (calcium and DAG-regulated)	4.8
8096704	NPNT	nephronectin	4.7
8127446	COL9A1	collagen, type IX, alpha 1	4.7
8055222	POTEF	POTE ankyrin domain family, member F	4.7
8041467	VIT	vitrin	4.6
8140955	CDK6	cyclin-dependent kinase 6	4.6
7947274	MPPED2	metallophosphoesterase domain containing 2	4.6
8047248	PLCL1	phospholipase C-like 1	4.5
8043687	ANKRD36C	ankyrin repeat domain 36C	4.5
8090133	CCDC14	coiled-coil domain containing 14	4.5
8036302	LOC100506930	uncharacterized LOC100506930	4.5
8128284	EPHA7	EPH receptor A7	4.4
8141140	DLX5	distal-less homeobox 5	4.4
8168589	ZNF711	zinc finger protein 711	4.4
7911241	OR2L8	olfactory receptor, family 2, subfamily L, member 8	4.4
8028924	MIA-RAB4B	MIA-RAB4B readthrough	4.4
7969288	OLFM4	olfactomedin 4	4.4
8147000	ZFHX4	zinc finger homeobox 4	4.3
7988444	MYEF2	myelin expression factor 2	4.3
7908924	PRELP	proline	4.3
8099982	APBB2	amyloid beta (A4) precursor protein-binding, family B, member 2	4.2
8054054	ANKRD36B	ankyrin repeat domain 36B	4.2
8111490	PRLR	prolactin receptor	4.2
8151684	MMP16	matrix metallopeptidase 16 (membrane-inserted)	4.2
8067305	SYCP2	synaptonemal complex protein 2	4.1
7913869	STMN1	stathmin 1	4.1
7932407	ST8SIA6	ST8 alpha-N-acetyl-neuraminide alpha-2,8-sialyltransferase 6	4.1
7945680	H19	H19, imprinted maternally expressed transcript (non-protein coding)	4.1
8046428	RAPGEF4	Rap guanine nucleotide exchange factor (GEF) 4	4.1
7946323	OR5P2	olfactory receptor, family 5, subfamily P, member 2	4.1

probeset_id	Symbol	Gene_Name	FC_T/N
8122202	MYB	v-myb myeloblastosis viral oncogene homolog (avian)	17.8
8051573	CDC42EP3	CDC42 effector protein (Rho GTPase binding) 3	4.1
7908816	LGR6	leucine-rich repeat containing G protein-coupled receptor 6	4.0
8136645	TAS2R4	taste receptor, type 2, member 4	4.0
7925525	CEP170	centrosomal protein 170kDa	4.0
8108724	PCDHB10	protocadherin beta 10	4.0
8005110	ZNF286A	zinc finger protein 286A	3.9
7938364	WEE1	WEE1 homolog (S. pombe)	3.9
8021946	COLEC12	collectin sub-family member 12	3.9
8106784	RASA1	RAS p21 protein activator (GTPase activating protein) 1	3.9
8144699	AY461701	Homo sapiens liver-related low express protein 1 (LRLE1) mRNA, complete cds.	3.8
8108744	PCDHB14	protocadherin beta 14	3.8
8043697	ANKRD36	ankyrin repeat domain 36	3.8
8150509	PLAT	plasminogen activator, tissue	3.8
8156199	DAPK1	death-associated protein kinase 1	3.8
7951140	LOC100131541	uncharacterized LOC100131541	3.8
7961514	MGP	matrix Gla protein	3.8
8171359	GPM6B	glycoprotein M6B	3.8
8179595	GABBR1	gamma-aminobutyric acid (GABA) B receptor, 1	3.7
7927681	BICC1	bicaudal C homolog 1 (Drosophila)	3.7
8076894	MLC1	megalencephalic leukoencephalopathy with subcortical cysts 1	3.7
7951217	MMP7	matrix metallopeptidase 7 (matrilysin, uterine)	3.7
8139977	STAG3L3	stromal antigen 3-like 3	3.7
7933437	PTPN20B	protein tyrosine phosphatase, non-receptor type 20B	3.7
8150318	FGFR1	fibroblast growth factor receptor 1	3.7
8052845	TIA1	TIA1 cytotoxic granule-associated RNA binding protein	3.6
8031157	TTYH1	tweety homolog 1 (Drosophila)	3.6
8142270	NRCAM	neuronal cell adhesion molecule	3.6
7963353	KRT81	keratin 81	3.6
8005231	FAM106A	family with sequence similarity 106, member A	3.6
8019831	CLUL1	clusterin-like 1 (retinal)	3.6
7912887	MFAP2	microfibrillar-associated protein 2	3.6
8115327	SPARC	secreted protein, acidic, cysteine-rich (osteonectin)	3.6
7997504	CDH13	cadherin 13, H-cadherin (heart)	3.5
8068833	PDE9A	phosphodiesterase 9A	3.5
8140196	STAG3L1	stromal antigen 3-like 1	3.5
8020321	AF090940	Homo sapiens clone HQ0644 PRO0644 mRNA, complete cds.	3.5
8157383	COL27A1	collagen, type XXVII, alpha 1	3.5
8064978	JAG1	jagged 1	3.5
8108683	PCDHB2	protocadherin beta 2	3.5
7938834	NAV2	neuron navigator 2	3.5
7900336	AY527403	Homo sapiens 10kDa eEF1A interacting protein mRNA, complete cds.	3.4
8165217	NOTCH1	notch 1	3.4

probeset_id	Symbol	Gene_Name	FC_T/N
8122202	MYB	v-myb myeloblastosis viral oncogene homolog (avian)	17.8
8146711	C8orf44	chromosome 8 open reading frame 44	3.4
7970033	COL4A2	collagen, type IV, alpha 2	3.4
7952451	LOC100130428	IGYY565	3.4
8047910	PTH2R	parathyroid hormone 2 receptor	3.4
8005679	CCDC144B	coiled-coil domain containing 144B (pseudogene)	3.4
7964665	DPY19L2	dpy-19-like 2 (C. elegans)	3.4
8039607	PEG3	paternally expressed 3	3.4
8084923	LOC401109	uncharacterized LOC401109	3.4
8140258	PMS2P6	postmeiotic segregation increased 2 pseudogene 6	3.3
8155754	MAMDC2	MAM domain containing 2	3.3
8130867	THBS2	thrombospondin 2	3.3
8120067	SLC25A27	solute carrier family 25, member 27	3.3
8140668	SEMA3A	sema domain, immunoglobulin domain (Ig), short basic domain, secreted, (semaphorin) 3A	3.3
7926545	PLXDC2	plexin domain containing 2	3.3
8005957	SNORD4B	small nucleolar RNA, C	3.3
8047487	FZD7	frizzled family receptor 7	3.3
8148000	AARD	alanine and arginine rich domain containing protein	3.3
7903188	PTBP2	polypyrimidine tract binding protein 2	3.3
8110666	TRIM52	tripartite motif containing 52	3.3
8112521	NAIP	NLR family, apoptosis inhibitory protein	3.3
8078461	FBXL2	F-box and leucine-rich repeat protein 2	3.3
8124166	MBOAT1	membrane bound O-acyltransferase domain containing 1	3.2
8108749	PCDHB18	protocadherin beta 18 pseudogene	3.2
7916984	MIR186	microRNA 186	3.2
8152453	TRPS1	trichorhinophalangeal syndrome I	3.2
8133309	PMS2P5	postmeiotic segregation increased 2 pseudogene 5	3.2
7912157	ERRFI1	ERBB receptor feedback inhibitor 1	3.2
7925257	LYST	lysosomal trafficking regulator	3.2
8078196	KCNH8	potassium voltage-gated channel, subfamily H (eag-related), member 8	3.2
7911252	OR2L2	olfactory receptor, family 2, subfamily L, member 2	3.2
8054831	EN1	engrailed homeobox 1	3.2
8140280	PMS2L2	postmeiotic segregation increased 2-like 2 pseudogene	3.2
8054020	FAM178B	family with sequence similarity 178, member B	3.2
8012464	LOC100128288	uncharacterized LOC100128288	3.2
8144726	TUSC3	tumor suppressor candidate 3	3.2
8113504	NREP	neuronal regeneration related protein homolog (rat)	3.2
7952986	WNK1	WNK lysine deficient protein kinase 1	3.1
8023646	BCL2	B-cell CLL	3.1
7982187	APBA2	amyloid beta (A4) precursor protein-binding, family A, member 2	3.1
8048411	TTLL4	tubulin tyrosine ligase-like family, member 4	3.1
8109159	MIR145	microRNA 145	3.1
8063536	TFAP2C	transcription factor AP-2 gamma (activating enhancer binding protein 2 gamma)	3.1

probeset_id	Symbol	Gene_Name	FC_T/N
8122202	MYB	v-myb myeloblastosis viral oncogene homolog (avian)	17.8
7967358	PITPNM2	phosphatidylinositol transfer protein, membrane-associated 2	3.1
8129273	C6orf170	chromosome 6 open reading frame 170	3.1
8139896	PMS2P4	postmeiotic segregation increased 2 pseudogene 4	3.1
8133106	SNORA22	small nucleolar RNA, H	3.1
7908861	OCR1	ovarian cancer-related protein 1	3.1
8091411	TM4SF1	transmembrane 4 L six family member 1	3.0
7972983	POTEG	POTE ankyrin domain family, member G	3.0
8006336	LRRC37B	leucine rich repeat containing 37B	3.0
8027297	ZNF738	zinc finger protein 738	3.0
8035793	ZNF737	zinc finger protein 737	3.0
7945110	ST3GAL4	ST3 beta-galactoside alpha-2,3-sialyltransferase 4	3.0
8138592	TRA2A	transformer 2 alpha homolog (Drosophila)	3.0
7902957	EPHX4	epoxide hydrolase 4	3.0
8108753	PCDHB15	protocadherin beta 15	3.0
8098439	EPCAM	epithelial cell adhesion molecule	3.0
8074286	MICAL3	microtubule associated monoxygenase, calponin and LIM domain containing 3	3.0
8081564	CD96	CD96 molecule	3.0
7982366	SCG5	secretogranin V (7B2 protein)	3.0
7924817	PRO2012	uncharacterized protein PRO2012	2.9
7972750	COL4A1	collagen, type IV, alpha 1	2.9
7938989	GAS2	growth arrest-specific 2	2.9
8145669	RBPMS	RNA binding protein with multiple splicing	2.9
8131263	SNORD13P2	small nucleolar RNA, C	2.9
8044225	SULT1C4	sulfotransferase family, cytosolic, 1C, member 4	2.9
8103695	MFAP3L	microfibrillar-associated protein 3-like	2.9
8083677	SCHIP1	schwannomin interacting protein 1	2.9
8053266	TACR1	tachykinin receptor 1	2.9
8133754	DTX2P1-UPK3BP1-[illegible]	DTX2P1-UPK3BP1-PMS2P11 readthrough (non-protein coding)	2.9
7996954	NFAT5	nuclear factor of activated T-cells 5, tonicity-responsive	2.9
8150978	CA8	carbonic anhydrase VIII	2.9
8102720	ANKRD50	ankyrin repeat domain 50	2.9
7968417	FRY	furry homolog (Drosophila)	2.9
8047659	ABI2	abl-interactor 2	2.9
8141395	MCM7	minichromosome maintenance complex component 7	2.9
8010978	LOC100130876	uncharacterized LOC100130876	2.8
8055143	LOC440905	uncharacterized LOC440905	2.8
7961291	TAS2R31	taste receptor, type 2, member 31	2.8
8070584	TMPRSS3	transmembrane protease, serine 3	2.8
8091402	TM4SF18	transmembrane 4 L six family member 18	2.8
7961285	TAS2R20	taste receptor, type 2, member 20	2.8
8157727	GPR21	G protein-coupled receptor 21	2.8
7963054	TUBA1A	tubulin, alpha 1a	2.8

probeset_id	Symbol	Gene_Name	FC_T/N
8122202	MYB	v-myb myeloblastosis viral oncogene homolog (avian)	17.8
8033818	OLFM2	olfactomedin 2	2.8
8147461	SDC2	syndecan 2	2.8
8001067	HERC2P4	hect domain and RLD 2 pseudogene 4	2.8
8116247	ZNF354A	zinc finger protein 354A	2.8
7911329	LOC100134822	uncharacterized LOC100134822	2.8
7916669	DOCK7	dedicator of cytokinesis 7	2.8
8084524	EPHB3	EPH receptor B3	2.8
8022424	NF1P3	neurofibromin 1 pseudogene 3	2.8
8137666	SEPT14	septin 14	2.8
7906307	KIRREL	kin of IRRE like (Drosophila)	2.8
8079021	CTNNB1	catenin (cadherin-associated protein), beta 1, 88kDa	2.8
8057959	PGAP1	post-GPI attachment to proteins 1	2.8
7976496	SERPINA3	serpin peptidase inhibitor, clade A (alpha-1 antiproteinase, antitrypsin), member 3	2.8
7969794	LOC100132099	FRSS1829	2.8
8083594	PTX3	pentraxin 3, long	2.8
7897449	SPSB1	splA	2.8
7981346	MOK	MOK protein kinase	2.8
8157153	PALM2-AKAP2	PALM2-AKAP2 readthrough	2.8
7947358	TPT1-AS1	TPT1 antisense RNA 1 (non-protein coding)	2.8
8045210	CYP4F43P	cytochrome P450, family 4, subfamily F, polypeptide 43, pseudogene	2.8
8151931	TSPYL5	TSPY-like 5	2.8
8079131	FAM198A	family with sequence similarity 198, member A	2.8
7925561	HNRNPU-AS1	HNRNPU antisense RNA 1 (non-protein coding)	2.8
8005225	USP32P1	ubiquitin specific peptidase 32 pseudogene 1	2.7
8175217	GPC4	glypican 4	2.7
7950391	PGM2L1	phosphoglucomutase 2-like 1	2.7
8173217	ARHGEF9	Cdc42 guanine nucleotide exchange factor (GEF) 9	2.7
7975416	PCNX	pecanex homolog (Drosophila)	2.7
8055016	AF234262	Homo sapiens IL-1beta-regulated neutrophil survival protein mRNA, complete cds.	2.7
8143663	EZH2	enhancer of zeste homolog 2 (Drosophila)	2.7
7994058	SCNN1G	sodium channel, non-voltage-gated 1, gamma subunit	2.7
8013262	USP32P2	ubiquitin specific peptidase 32 pseudogene 2	2.7
8108720	PCDHB9	protocadherin beta 9	2.7
7923453	KDM5B	lysine (K)-specific demethylase 5B	2.7
7951159	RN5S346	RNA, 5S ribosomal 346	2.7
8132318	ANLN	anillin, actin binding protein	2.7
8161632	PTAR1	protein prenyltransferase alpha subunit repeat containing 1	2.7
8141490	PMS2P1	postmeiotic segregation increased 2 pseudogene 1	2.7
7917676	GLMN	glomulin, FKBP associated protein	2.7
8177834	ATAT1	alpha tubulin acetyltransferase 1	2.7
8001099	HERC2	HECT and RLD domain containing E3 ubiquitin protein ligase 2	2.7
7902441	ST6GALNAC5	ST6 (alpha-N-acetyl-neuraminyl-2,3-beta-galactosyl-1,3)-N-acetylgalactosaminide alpha-2,6- [illegible]	2.7

probeset_id	Symbol	Gene_Name	FC_T/N
8122202	MYB	v-myb myeloblastosis viral oncogene homolog (avian)	17.8
7896754	LOC100287497	uncharacterized LOC100287497	2.7
8134789	PILRB	paired immunoglobin-like type 2 receptor beta	2.7
8035782	ZNF682	zinc finger protein 682	2.7
8118890	SCUBE3	signal peptide, CUB domain, EGF-like 3	2.7
7950042	SHANK2	SH3 and multiple ankyrin repeat domains 2	2.7
7917649	TGFBR3	transforming growth factor, beta receptor III	2.7
7948904	SNORD28	small nucleolar RNA, C	2.7
7951036	TAF1D	TATA box binding protein (TBP)-associated factor, RNA polymerase I, D, 41kDa	2.7
7991224	HAPLN3	hyaluronan and proteoglycan link protein 3	2.6
8123446	SMOC2	SPARC related modular calcium binding 2	2.6
8144036	XRCC2	X-ray repair complementing defective repair in Chinese hamster cells 2	2.6
8130891	WDR27	WD repeat domain 27	2.6
7903404	RNPC3	RNA-binding region (RNP1, RRM) containing 3	2.6
8133914	DMTF1	cyclin D binding myb-like transcription factor 1	2.6
8104141	PLEKHG4B	pleckstrin homology domain containing, family G (with RhoGef domain) member 4B	2.6
7970624	CENPJ	centromere protein J	2.6
8090152	ROPN1B	rhophilin associated tail protein 1B	2.6
8043114	TCF7L1	transcription factor 7-like 1 (T-cell specific, HMG-box)	2.6
7961281	TAS2R50	taste receptor, type 2, member 50	2.6
8139832	ZNF117	zinc finger protein 117	2.6
7972003	KLF12	Kruppel-like factor 12	2.6
8088636	LOC100508226	HHSL751	2.6
8074780	YPEL1	yippee-like 1 (Drosophila)	2.6
7945663	IFITM10	interferon induced transmembrane protein 10	2.6
8046380	ITGA6	integrin, alpha 6	2.6
8031857	ZNF135	zinc finger protein 135	2.6
7951133	MAML2	mastermind-like 2 (Drosophila)	2.6
8091600	PLCH1	phospholipase C, eta 1	2.6
8076113	FAM227A	family with sequence similarity 227, member A	2.6
7937772	IGF2	insulin-like growth factor 2 (somatomedin A)	2.6
8069574	C21orf91	chromosome 21 open reading frame 91	2.6
7902977	KIAA1107	KIAA1107	2.6
7936734	FGFR2	fibroblast growth factor receptor 2	2.6
7943369	TMEM133	transmembrane protein 133	2.6
7939374	AF274942	Homo sapiens PNAS-17 mRNA, complete cds.	2.6
8156905	TMEFF1	transmembrane protein with EGF-like and two follistatin-like domains 1	2.6
7933263	PTPN20A	protein tyrosine phosphatase, non-receptor type 20A	2.6
8001423	RPGRIP1L	RPGRIP1-like	2.6
8155192	GLIPR2	GLI pathogenesis-related 2	2.6
8115196	ZNF300	zinc finger protein 300	2.6
8154394	SNAPC3	small nuclear RNA activating complex, polypeptide 3, 50kDa	2.6
8102232	LEF1	lymphoid enhancer-binding factor 1	2.6

probeset_id	Symbol	Gene_Name	FC_T/N
8122202	MYB	v-myb myeloblastosis viral oncogene homolog (avian)	17.8
8104131	MGC39584	uncharacterized LOC441058	2.6
8114970	C5orf46	chromosome 5 open reading frame 46	2.6
8127932	TBX18	T-box 18	2.6
8021442	ZNF532	zinc finger protein 532	2.5
8074925	GUSBP11	glucuronidase, beta pseudogene 11	2.5
8117165	SOX4	SRY (sex determining region Y)-box 4	2.5
7961339	LRP6	low density lipoprotein receptor-related protein 6	2.5
7902891	ZNF326	zinc finger protein 326	2.5
7953981	ETV6	ets variant 6	2.5
8143188	CREB3L2	cAMP responsive element binding protein 3-like 2	2.5
8095080	PDGFRA	platelet-derived growth factor receptor, alpha polypeptide	2.5
8169949	MST4	serine	2.5
7910618	SLC35F3	solute carrier family 35, member F3	2.5
8012257	TP53	tumor protein p53	2.5
8105144	AK001108	Homo sapiens cDNA FLJ10246 fis, clone HEMBB1000673.	2.5
8082767	TMEM108	transmembrane protein 108	2.5
8094574	TBC1D1	TBC1 (tre-2	2.5
7985560	CSPG4P5	chondroitin sulfate proteoglycan 4 pseudogene 5	2.5
8018114	SDK2	sidekick cell adhesion molecule 2	2.5
8156164	KIF27	kinesin family member 27	2.5
8105663	NLN	neurolysin (metallopeptidase M3 family)	2.5
7924712	LIN9	lin-9 homolog (C. elegans)	2.5
8112615	ENC1	ectodermal-neural cortex 1 (with BTB-like domain)	2.5
7991598	SNRPA1	small nuclear ribonucleoprotein polypeptide A'	2.5
8000687	LOC595101	smg-1 homolog, phosphatidylinositol 3-kinase-related kinase (C. elegans) pseudogene	2.5
7903407	AMY2B	amylase, alpha 2B (pancreatic)	2.5
8047097	GLS	glutaminase	2.5
8008493	LUC7L3	LUC7-like 3 (S. cerevisiae)	2.5
8028194	ZNF382	zinc finger protein 382	2.5
7950082	LOC100133315	transient receptor potential cation channel, subfamily C, member 2-like	2.5
7960143	ZNF84	zinc finger protein 84	2.5
8000823	SMG1	smg-1 homolog, phosphatidylinositol 3-kinase-related kinase (C. elegans)	2.5
8084739	FLJ42393	uncharacterized LOC401105	2.5
8104139	LOC389834	ankyrin repeat domain 57 pseudogene	2.5
7905918	EFNA3	ephrin-A3	2.5
8136647	TAS2R5	taste receptor, type 2, member 5	2.5
8012475	MYH10	myosin, heavy chain 10, non-muscle	2.5
8123864	TFAP2A	transcription factor AP-2 alpha (activating enhancer binding protein 2 alpha)	2.5
7972888	PCID2	PCI domain containing 2	2.5
8005765	WSB1	WD repeat and SOCS box containing 1	2.5
8022612	ZNF521	zinc finger protein 521	2.5
8056047	WDSUB1	WD repeat, sterile alpha motif and U-box domain containing 1	2.5

probeset_id	Symbol	Gene_Name	FC_T/N
8122202	MYB	v-myb myeloblastosis viral oncogene homolog (avian)	17.8
7969736	FARP1	FERM, RhoGEF (ARHGEF) and pleckstrin domain protein 1 (chondrocyte-derived)	2.5
7946326	OR5P3	olfactory receptor, family 5, subfamily P, member 3	2.5
7993999	LOC100271836	smg-1 homolog, phosphatidylinositol 3-kinase-related kinase (C. elegans) pseudogene	2.4
8000834	LOC440354	smg-1 homolog, phosphatidylinositol 3-kinase-related kinase (C. elegans) pseudogene	2.4
7961757	ST8SIA1	ST8 alpha-N-acetyl-neuraminide alpha-2,8-sialyltransferase 1	2.4
7961540	RERG	RAS-like, estrogen-regulated, growth inhibitor	2.4
8099259	AFAP1	actin filament associated protein 1	2.4
8058390	RAPH1	Ras association (RalGDS	2.4
8096875	ENPEP	glutamyl aminopeptidase (aminopeptidase A)	2.4
7961279	TAS2R14	taste receptor, type 2, member 14	2.4
8000156	SMG1P1	smg-1 homolog, phosphatidylinositol 3-kinase-related kinase pseudogene 1	2.4
7983638	DTWD1	DTW domain containing 1	2.4
8097058	CEP170P1	centrosomal protein 170kDa pseudogene 1	2.4
8104568	LOC100133299	GALI1870	2.4
8170420	MAMLD1	mastermind-like domain containing 1	2.4
8147079	LRRCC1	leucine rich repeat and coiled-coil centrosomal protein 1	2.4
8027241	ZNF253	zinc finger protein 253	2.4
7986701	HERC2P2	hect domain and RLD 2 pseudogene 2	2.4
8122773	MTHFD1L	methylenetetrahydrofolate dehydrogenase (NADP+ dependent) 1-like	2.4
7986383	IGF1R	insulin-like growth factor 1 receptor	2.4
8071061	TPTEP1	transmembrane phosphatase with tensin homology pseudogene 1	2.4
8123520	LINC00266-1	long intergenic non-protein coding RNA 266-1	2.4
7991777	C4orf46	chromosome 4 open reading frame 46	2.4
7947189	CCDC34	coiled-coil domain containing 34	2.4
8035847	ZNF675	zinc finger protein 675	2.4
7925691	ZNF124	zinc finger protein 124	2.4
8017262	BRIP1	BRCA1 interacting protein C-terminal helicase 1	2.4
7991047	LOC100131860	uncharacterized LOC100131860	2.4
8174119	ZMAT1	zinc finger, matrin-type 1	2.4
7908347	OCLM	oculomedin	2.4
7927876	TET1	tet methylcytosine dioxygenase 1	2.4
7970329	GAS6	growth arrest-specific 6	2.4
8147101	E2F5	E2F transcription factor 5, p130-binding	2.4
7998174	LUC7L	LUC7-like (S. cerevisiae)	2.4
7978997	MAP4K5	mitogen-activated protein kinase kinase kinase kinase 5	2.4
8103644	AB062480	Homo sapiens OK	2.4
8135585	AY143171	Homo sapiens testin-related protein TRG mRNA, complete cds.	2.4
8036324	ZNF260	zinc finger protein 260	2.4
8052399	BCL11A	B-cell CLL	2.4
7965486	CCDC41	coiled-coil domain containing 41	2.4
8062623	PLCG1	phospholipase C, gamma 1	2.4
8136347	CALD1	caldesmon 1	2.4

probeset_id	Symbol	Gene_Name	FC_T/N
8122202	MYB	v-myb myeloblastosis viral oncogene homolog (avian)	17.8
8170648	BGN	biglycan	2.4
7962112	CAPRIN2	caprin family member 2	2.4
7951144	CCDC82	coiled-coil domain containing 82	2.4
8089701	ZBTB20	zinc finger and BTB domain containing 20	2.4
8016789	MBTD1	mbt domain containing 1	2.4
7954717	BICD1	bicaudal D homolog 1 (Drosophila)	2.4
7961287	TAS2R19	taste receptor, type 2, member 19	2.4
8079079	NKTR	natural killer-tumor recognition sequence	2.4
7901993	CACHD1	cache domain containing 1	2.4
8139840	ERV3-1	endogenous retrovirus group 3, member 1	2.4
8084219	KLHL24	kelch-like 24 (Drosophila)	2.4
8038347	TEAD2	TEA domain family member 2	2.4
7916969	ZRANB2	zinc finger, RAN-binding domain containing 2	2.3
8093332	ZNF876P	zinc finger protein 876, pseudogene	2.3
8109938	RANBP17	RAN binding protein 17	2.3
8137670	PDGFA	platelet-derived growth factor alpha polypeptide	2.3
8147112	CA13	carbonic anhydrase XIII	2.3
8066347	PTPRT	protein tyrosine phosphatase, receptor type, T	2.3
8178059	LY6G5B	lymphocyte antigen 6 complex, locus G5B	2.3
8022320	NPIPL2	nuclear pore complex interacting protein-like 2	2.3
7972682	KDELC1	KDEL (Lys-Asp-Glu-Leu) containing 1	2.3
8108716	PCDHB16	protocadherin beta 16	2.3
7932132	FRMD4A	FERM domain containing 4A	2.3
8047174	SLC39A10	solute carrier family 39 (zinc transporter), member 10	2.3
8176865	PCMTD2	protein-L-isoaspartate (D-aspartate) O-methyltransferase domain containing 2	2.3
8009255	CEP95	centrosomal protein 95kDa	2.3
7918925	TRIM45	tripartite motif containing 45	2.3
8120271	FBXO9	F-box protein 9	2.3
8152703	FBXO32	F-box protein 32	2.3
7943160	SCARNA9L	small Cajal body-specific RNA 9-like	2.3
8027368	ZNF254	zinc finger protein 254	2.3
8147313	TMEM67	transmembrane protein 67	2.3
8128394	PNISR	PNN-interacting serine	2.3
8052598	WDPCP	WD repeat containing planar cell polarity effector	2.3
8135211	FAM185A	family with sequence similarity 185, member A	2.3
7945864	ZNF195	zinc finger protein 195	2.3
7922402	GAS5	growth arrest-specific 5 (non-protein coding)	2.3
7997239	LOC100507607	nuclear pore complex-interacting protein-like 2-like	2.3
8064637	C20orf194	chromosome 20 open reading frame 194	2.3
7973056	APEX1	APEX nuclease (multifunctional DNA repair enzyme) 1	2.3
8094476	TBC1D19	TBC1 domain family, member 19	2.3
8112560	SMA5	glucuronidase, beta pseudogene	2.3

probeset_id	Symbol	Gene_Name	FC_T/N
8122202	MYB	v-myb myeloblastosis viral oncogene homolog (avian)	17.8
8053315	LRRTM4	leucine rich repeat transmembrane neuronal 4	2.3
8099612	GPR125	G protein-coupled receptor 125	2.3
8037762	CCDC8	coiled-coil domain containing 8	2.3
8084232	YEATS2	YEATS domain containing 2	2.3
8105991	SMA4	glucuronidase, beta pseudogene	2.3
8082165	KALRN	kalirin, RhoGEF kinase	2.3
8072705	RASD2	RASD family, member 2	2.3
8088560	ADAMTS9	ADAM metallopeptidase with thrombospondin type 1 motif, 9	2.3
8170179	VGLL1	vestigial like 1 (Drosophila)	2.3
8119423	ADCY10P1	adenylate cyclase 10 (soluble) pseudogene 1	2.3
7930537	TCF7L2	transcription factor 7-like 2 (T-cell specific, HMG-box)	2.3
8153262	SLC45A4	solute carrier family 45, member 4	2.3
7981943	SNORD64	small nucleolar RNA, C	2.3
8148317	MYC	v-myc myelocytomatosis viral oncogene homolog (avian)	2.3
7896744	OR4F3	olfactory receptor, family 4, subfamily F, member 3	2.3
8091550	KIAA1328	KIAA1328	2.3
8166104	OFD1	oral-facial-digital syndrome 1	2.3
8155591	ANKRD20A8P	ankyrin repeat domain 20 family, member A8, pseudogene	2.3
7915787	PIK3R3	phosphoinositide-3-kinase, regulatory subunit 3 (gamma)	2.3
7993580	PKD1P1	polycystic kidney disease 1 (autosomal dominant) pseudogene 1	2.3
8159876	RFX3	regulatory factor X, 3 (influences HLA class II expression)	2.3
7994026	LOC100132247	nuclear pore complex interacting protein related gene	2.3
8104621	GUSBP1	glucuronidase, beta pseudogene 1	2.3
8009040	MRC2	mannose receptor, C type 2	2.3
8117395	HIST1H2BF	histone cluster 1, H2bf	2.3
8045736	FMNL2	formin-like 2	2.3
8171493	CTPS2	CTP synthase 2	2.3
8136641	TAS2R3	taste receptor, type 2, member 3	2.3
8110478	ZNF454	zinc finger protein 454	2.3
8102532	PDE5A	phosphodiesterase 5A, cGMP-specific	2.3
7909568	DTL	denticleless E3 ubiquitin protein ligase homolog (Drosophila)	2.3
8112376	CENPK	centromere protein K	2.3
8160138	NFIB	nuclear factor I	2.2
8140709	KIAA1324L	KIAA1324-like	2.2
8145611	FZD3	frizzled family receptor 3	2.2
8132917	ZNF713	zinc finger protein 713	2.2
7920852	KIAA0907	KIAA0907	2.2
8035813	ZNF43	zinc finger protein 43	2.2
8048171	PKI55	DKFZp434H1419	2.2
8041813	CRIPT	cysteine-rich PDZ-binding protein	2.2
8047738	NRP2	neuropilin 2	2.2
8046461	ZAK	sterile alpha motif and leucine zipper containing kinase AZK	2.2

probeset_id	Symbol	Gene_Name	FC_T/N
8122202	MYB	v-myb myeloblastosis viral oncogene homolog (avian)	17.8
7900051	EIF2C3	eukaryotic translation initiation factor 2C, 3	2.2
7943442	DYNC2H1	dynein, cytoplasmic 2, heavy chain 1	2.2
8060736	BC008667	Homo sapiens cDNA clone MGC:17708 IMAGE:3868595, complete cds.	2.2
8130071	C15orf29	chromosome 15 open reading frame 29	2.2
7974341	GNG2	guanine nucleotide binding protein (G protein), gamma 2	2.2
8141882	DPY19L2P2	dpy-19-like 2 pseudogene 2 (C. elegans)	2.2
7932433	NSUN6	NOP2	2.2
8111153	MYO10	myosin X	2.2
8048381	STK36	serine	2.2
7934706	AF130084	Homo sapiens clone FLB8310 PRO2225 mRNA, complete cds.	2.2
7933877	JMJD1C	jumonji domain containing 1C	2.2
7967210	LOC338799	uncharacterized LOC338799	2.2
7985053	FBXO22	F-box protein 22	2.2
8087201	IP6K2	inositol hexakisphosphate kinase 2	2.2
8124531	HIST1H3I	histone cluster 1, H3i	2.2
8148966	RPL23AP53	ribosomal protein L23a pseudogene 53	2.2
7902127	SGIP1	SH3-domain GRB2-like (endophilin) interacting protein 1	2.2
8069178	ADARB1	adenosine deaminase, RNA-specific, B1	2.2
8050497	OSR1	odd-skipped related 1 (Drosophila)	2.2
8022295	PIEZO2	piezo-type mechanosensitive ion channel component 2	2.2
8037767	PNMAL1	paraneoplastic Ma antigen family-like 1	2.2
8132118	AQP1	aquaporin 1 (Colton blood group)	2.2
7957008	CPSF6	cleavage and polyadenylation specific factor 6, 68kDa	2.2
7925904	AKR1E2	aldo-keto reductase family 1, member E2	2.2
7927353	AGAP9	ArfGAP with GTPase domain, ankyrin repeat and PH domain 9	2.2
8118322	SNORD52	small nucleolar RNA, C	2.2
7987248	GOLGA8A	golgin A8 family, member A	2.2
8095545	RUFY3	RUN and FYVE domain containing 3	2.2
8069644	APP	amyloid beta (A4) precursor protein	2.2
7933092	ZNF248	zinc finger protein 248	2.2
8102171	TBCK	TBC1 domain containing kinase	2.2
7972369	UGGT2	UDP-glucose glycoprotein glucosyltransferase 2	2.2
7942596	SERPINH1	serpin peptidase inhibitor, clade H (heat shock protein 47), member 1, (collagen binding [illegible]	2.2
8092251	GNB4	guanine nucleotide binding protein (G protein), beta polypeptide 4	2.2
8161575	CBWD5	COBW domain containing 5	2.2
7953765	RIMKLB	ribosomal modification protein rimK-like family member B	2.2
7943349	ARHGAP42	Rho GTPase activating protein 42	2.2
7930559	AK027209	Homo sapiens cDNA: FLJ23556 fis, clone LNG09443.	2.2
7917976	SASS6	spindle assembly 6 homolog (C. elegans)	2.2
7934411	USP54	ubiquitin specific peptidase 54	2.2
7921916	RGS5	regulator of G-protein signaling 5	2.2
8147503	LAPTM4B	lysosomal protein transmembrane 4 beta	2.2

probeset_id	Symbol	Gene_Name	FC_T/N
8122202	MYB	v-myb myeloblastosis viral oncogene homolog (avian)	17.8
8001800	CDH11	cadherin 11, type 2, OB-cadherin (osteoblast)	2.2
7961249	TAS2R10	taste receptor, type 2, member 10	2.2
8108954	TCERG1	transcription elongation regulator 1	2.2
8005839	TMEM97	transmembrane protein 97	2.2
8086908	PLXNB1	plexin B1	2.2
8154416	CCDC171	coiled-coil domain containing 171	2.2
8166355	CNKSR2	connector enhancer of kinase suppressor of Ras 2	2.2
7932390	TRDMT1	tRNA aspartic acid methyltransferase 1	2.2
8120335	FAM83B	family with sequence similarity 83, member B	2.2
7920000	POGZ	pogo transposable element with ZNF domain	2.2
7897803	PLOD1	procollagen-lysine, 2-oxoglutarate 5-dioxygenase 1	2.2
7910915	CHRM3	cholinergic receptor, muscarinic 3	2.2
8118228	CSNK2B	casein kinase 2, beta polypeptide	2.2
8038967	ZNF83	zinc finger protein 83	2.2
8111339	MTMR12	myotubularin related protein 12	2.2
7960947	A2M	alpha-2-macroglobulin	2.2
8178090	C6orf48	chromosome 6 open reading frame 48	2.2
8085145	RAD18	RAD18 homolog (S. cerevisiae)	2.2
7977472	OR11H12	olfactory receptor, family 11, subfamily H, member 12	2.2
8124691	HCG8	HLA complex group 8	2.2
8126710	SUPT3H	suppressor of Ty 3 homolog (S. cerevisiae)	2.2
8162490	HIATL1	hippocampus abundant transcript-like 1	2.2
7925500	CHML	choroideremia-like (Rab escort protein 2)	2.2
8028186	ZNF146	zinc finger protein 146	2.1
7951046	MRE11A	MRE11 meiotic recombination 11 homolog A (S. cerevisiae)	2.1
8000205	NPIPL3	nuclear pore complex interacting protein-like 3	2.1
8066254	LOC388796	uncharacterized LOC388796	2.1
8166511	PDK3	pyruvate dehydrogenase kinase, isozyme 3	2.1
8040985	ZNF512	zinc finger protein 512	2.1
7923426	UBE2T	ubiquitin-conjugating enzyme E2T (putative)	2.1
7999614	LOC399491	GPS, PLAT and transmembrane domain-containing protein	2.1
8169419	ALG13	asparagine-linked glycosylation 13 homolog (S. cerevisiae)	2.1
8020308	C18orf1	chromosome 18 open reading frame 1	2.1
8122365	GPR126	G protein-coupled receptor 126	2.1
7905909	EFNA4	ephrin-A4	2.1
8070194	RUNX1	runt-related transcription factor 1	2.1
8015607	STAT3	signal transducer and activator of transcription 3 (acute-phase response factor)	2.1
7933139	ZNF33B	zinc finger protein 33B	2.1
8048014	RPE	ribulose-5-phosphate-3-epimerase	2.1
8058477	KLF7	Kruppel-like factor 7 (ubiquitous)	2.1
8010078	LOC100507246	uncharacterized LOC100507246	2.1
7922408	SNORD78	small nucleolar RNA, C	2.1

probeset_id	Symbol	Gene_Name	FC_T/N
8122202	MYB	v-myb myeloblastosis viral oncogene homolog (avian)	17.8
8021181	SCARNA17	small Cajal body-specific RNA 17	2.1
7994565	RRN3P2	RNA polymerase I transcription factor homolog (S. cerevisiae) pseudogene 2	2.1
8093453	FLJ35816	FLJ35816 protein	2.1
8015526	KAT2A	K(lysine) acetyltransferase 2A	2.1
7987369	ATPBD4	ATP binding domain 4	2.1
7943690	DDX10	DEAD (Asp-Glu-Ala-Asp) box polypeptide 10	2.1
8158998	RPL7A	ribosomal protein L7a	2.1
8039593	ZNF667	zinc finger protein 667	2.1
7965523	NR2C1	nuclear receptor subfamily 2, group C, member 1	2.1
8137474	ACTR3B	ARP3 actin-related protein 3 homolog B (yeast)	2.1
8094342	PACRGL	PARK2 co-regulated-like	2.1
8035803	ZNF708	zinc finger protein 708	2.1
7933413	BMS1P5	BMS1 pseudogene 5	2.1
7969204	WDFY2	WD repeat and FYVE domain containing 2	2.1
8057045	FKBP7	FK506 binding protein 7	2.1
7902883	LRRC8D	leucine rich repeat containing 8 family, member D	2.1
8025488	ZNF559-ZNF177	ZNF559-ZNF177 readthrough	2.1
8176026	FLNA	filamin A, alpha	2.1
8173310	OPHN1	oligophrenin 1	2.1
8113433	EFNA5	ephrin-A5	2.1
7920877	ARHGEF2	Rho	2.1
8047577	ALS2CR8	amyotrophic lateral sclerosis 2 (juvenile) chromosome region, candidate 8	2.1
7943890	LOC100132686	uncharacterized LOC100132686	2.1
7918913	IGSF3	immunoglobulin superfamily, member 3	2.1
7994371	LOC728741	uncharacterized LOC728741	2.1
7961960	RN5S354	RNA, 5S ribosomal 354	2.1
8068761	ABCG1	ATP-binding cassette, sub-family G (WHITE), member 1	2.1
8162850	TEX10	testis expressed 10	2.1
8141625	EPHB4	EPH receptor B4	2.1
7959761	FAM101A	family with sequence similarity 101, member A	2.1
8021924	THOC1	THO complex 1	2.1
8161373	Rinn lincRNA	linc-FAM75A7-2 chr9:-:42363047-42367092	2.1
8050869	AY358728	Homo sapiens clone DNA108758 GNNC2999 (UNQ2999) mRNA, complete cds.	2.1
8111952	C5orf28	chromosome 5 open reading frame 28	2.1
8133049	ZNF107	zinc finger protein 107	2.1
7949956	MTL5	metallothionein-like 5, testis-specific (tesmin)	2.1
8167673	MAGED4B	melanoma antigen family D, 4B	2.1
8007799	MGC57346	uncharacterized LOC401884	2.1
8013015	CENPV	centromere protein V	2.1
7933427	AGAP8	ArfGAP with GTPase domain, ankyrin repeat and PH domain 8	2.1
8088264	IL17RD	interleukin 17 receptor D	2.1
8088142	CHDH	choline dehydrogenase	2.1

probeset_id	Symbol	Gene_Name	FC_T/N
8122202	MYB	v-myb myeloblastosis viral oncogene homolog (avian)	17.8
7980940	ATXN3	ataxin 3	2.1
7993404	NPIP	nuclear pore complex interacting protein	2.1
8023377	MEX3C	mex-3 homolog C (C. elegans)	2.1
7916024	TTC39A	tetratricopeptide repeat domain 39A	2.1
8108697	PCDHB5	protocadherin beta 5	2.1
8141872	NAPEPLD	N-acyl phosphatidylethanolamine phospholipase D	2.1
8156263	SPIN1	spindlin 1	2.1
8090737	NPHP3	nephronophthisis 3 (adolescent)	2.1
7970793	SLC46A3	solute carrier family 46, member 3	2.1
8006237	LOC400590	uncharacterized LOC400590	2.1
8141371	GJC3	gap junction protein, gamma 3, 30.2kDa	2.1
8161587	CBWD3	COBW domain containing 3	2.1
8027260	ZNF486	zinc finger protein 486	2.1
8147221	OSGIN2	oxidative stress induced growth inhibitor family member 2	2.1
8133057	ZNF138	zinc finger protein 138	2.1
7959408	KNTC1	kinetochore associated 1	2.1
8175432	RBMX	RNA binding motif protein, X-linked	2.1
8160040	PTPRD	protein tyrosine phosphatase, receptor type, D	2.1
8131519	PHF14	PHD finger protein 14	2.1
8166469	SAT1	spermidine	2.1
8123155	PNLDC1	poly(A)-specific ribonuclease (PARN)-like domain containing 1	2.1
7984569	LRRC49	leucine rich repeat containing 49	2.1
8094134	LOC728419	ubiquitin carboxyl-terminal hydrolase 17-like	2.1
8031992	LOC100506479	uncharacterized LOC100506479	2.1
8014974	TOP2A	topoisomerase (DNA) II alpha 170kDa	2.1
8056113	LY75	lymphocyte antigen 75	2.1
7909503	SERTAD4	SERTA domain containing 4	2.1
8088348	FAM116A	family with sequence similarity 116, member A	2.1
8171297	MID1	midline 1 (Opitz	2.1
8161433	AK091508	Homo sapiens cDNA FLJ34189 fis, clone FCBBF3017535.	2.1
8119858	POLH	polymerase (DNA directed), eta	2.1
7958000	CHPT1	choline phosphotransferase 1	2.1
8019737	KPNA2	karyopherin alpha 2 (RAG cohort 1, importin alpha 1)	2.1
8052908	CLEC4F	C-type lectin domain family 4, member F	2.1
7899562	PTPRU	protein tyrosine phosphatase, receptor type, U	2.1
8169617	PGRMC1	progesterone receptor membrane component 1	2.0
7971039	FAM48A	family with sequence similarity 48, member A	2.0
8139212	GLI3	GLI family zinc finger 3	2.0
8146717	SGK3	serum	2.0
8132439	C7orf10	chromosome 7 open reading frame 10	2.0
8075673	RBFOX2	RNA binding protein, fox-1 homolog (C. elegans) 2	2.0
8122099	ENPP1	ectonucleotide pyrophosphatase	2.0

probeset_id	Symbol	Gene_Name	FC_T/N
8122202	MYB	v-myb myeloblastosis viral oncogene homolog (avian)	17.8
7909708	CENPF	centromere protein F, 350	2.0
8105585	RNF180	ring finger protein 180	2.0
8070046	GCFC1	GC-rich sequence DNA-binding factor 1	2.0
8027247	ZNF93	zinc finger protein 93	2.0
8090988	CEP70	centrosomal protein 70kDa	2.0
8166289	CDKL5	cyclin-dependent kinase-like 5	2.0
8027304	ZNF493	zinc finger protein 493	2.0
8047606	NBEAL1	neurobeachin-like 1	2.0
8060977	C20orf94	chromosome 20 open reading frame 94	2.0
8117646	ZNF192	zinc finger protein 192	2.0
7968274	PAN3	PAN3 poly(A) specific ribonuclease subunit homolog (S. cerevisiae)	2.0
7948906	SNHG1	small nucleolar RNA host gene 1 (non-protein coding)	2.0
8165888	AF118077	Homo sapiens PRO1808 mRNA, complete cds.	2.0
7964347	TMEM194A	transmembrane protein 194A	2.0
8044346	LOC151009	uncharacterized LOC151009	2.0
7933574	AGAP4	ArfGAP with GTPase domain, ankyrin repeat and PH domain 4	2.0
8080714	FLNB	filamin B, beta	2.0
8153890	ZNF251	zinc finger protein 251	2.0
8160559	DDX58	DEAD (Asp-Glu-Ala-Asp) box polypeptide 58	2.0
8129418	PTPRK	protein tyrosine phosphatase, receptor type, K	2.0
8047538	BMPR2	bone morphogenetic protein receptor, type II (serine	2.0
8042601	ZNF638	zinc finger protein 638	2.0
8096463	SMARCAD1	SWI	2.0
8078784	XYLB	xylulokinase homolog (H. influenzae)	2.0
8059279	EPHA4	EPH receptor A4	2.0
8168316	OGT	O-linked N-acetylglucosamine (GlcNAc) transferase	2.0
7917433	ODF2L	outer dense fiber of sperm tails 2-like	2.0
7925978	FAM208B	family with sequence similarity 208, member B	2.0
7904478	LINC00328	long intergenic non-protein coding RNA 328	2.0
8091656	METTL15	methyltransferase like 15	2.0
8035842	ZNF91	zinc finger protein 91	2.0
8042195	AHSA2	AHA1, activator of heat shock 90kDa protein ATPase homolog 2 (yeast)	2.0
8149774	LOXL2	lysyl oxidase-like 2	2.0
8142036	SRPK2	SRSF protein kinase 2	2.0
8167835	TRO	trophinin	2.0
7920317	ILF2	interleukin enhancer binding factor 2, 45kDa	2.0
7916185	ZCCHC11	zinc finger, CCHC domain containing 11	2.0
7967091	SPPL3	signal peptide peptidase like 3	2.0
8066303	CHD6	chromodomain helicase DNA binding protein 6	2.0
8052526	XPO1	exportin 1 (CRM1 homolog, yeast)	2.0
7926896	CKS1B	CDC28 protein kinase regulatory subunit 1B	2.0
8035779	ZNF626	zinc finger protein 626	2.0

probeset_id	Symbol	Gene_Name	FC_T/N
8122202	MYB	v-myb myeloblastosis viral oncogene homolog (avian)	17.8
7968800	DGKH	diacylglycerol kinase, eta	2.0
7902992	RPAP2	RNA polymerase II associated protein 2	2.0
8112202	PLK2	polo-like kinase 2	2.0
8097262	SPATA5	spermatogenesis associated 5	2.0
8041644	PLEKHH2	pleckstrin homology domain containing, family H (with MyTH4 domain) member 2	2.0
8086141	EPM2AIP1	EPM2A (laforin) interacting protein 1	2.0
7928291	CHST3	carbohydrate (chondroitin 6) sulfotransferase 3	2.0
8128522	HACE1	HECT domain and ankyrin repeat containing E3 ubiquitin protein ligase 1	2.0
8006170	LRRC37BP1	leucine rich repeat containing 37B pseudogene 1	2.0
7919584	HIST2H2BF	histone cluster 2, H2bf	2.0
8064322	C20orf96	chromosome 20 open reading frame 96	2.0
7926189	SEC61A2	Sec61 alpha 2 subunit (S. cerevisiae)	2.0
8036151	HSPB6	heat shock protein, alpha-crystallin-related, B6	0.5
7947512	PAMR1	peptidase domain containing associated with muscle regeneration 1	0.5
8026047	JUNB	jun B proto-oncogene	-2.0
8002778	MLKL	mixed lineage kinase domain-like	-2.0
8091799	SPTSSB	serine palmitoyltransferase, small subunit B	-2.0
8172425	SLC38A5	solute carrier family 38, member 5	-2.0
8126382	C6orf132	chromosome 6 open reading frame 132	-2.0
7991386	CIB1	calcium and integrin binding 1 (calmyrin)	-2.0
8165496	TUBB4B	tubulin, beta 4B class IVb	-2.0
8137783	TMEM184A	transmembrane protein 184A	-2.0
8101874	ADH1A	alcohol dehydrogenase 1A (class I), alpha polypeptide	-2.0
7922474	KIAA0040	KIAA0040	-2.0
8161745	RN5S285	RNA, 5S ribosomal 285	-2.0
8028984	CYP2F1	cytochrome P450, family 2, subfamily F, polypeptide 1	-2.0
8129837	IL20RA	interleukin 20 receptor, alpha	-2.0
7901613	ACOT11	acyl-CoA thioesterase 11	-2.0
8061564	ID1	inhibitor of DNA binding 1, dominant negative helix-loop-helix protein	-2.0
7965873	IGF1	insulin-like growth factor 1 (somatomedin C)	-2.0
7917875	F3	coagulation factor III (thromboplastin, tissue factor)	-2.0
7966779	NOS1	nitric oxide synthase 1 (neuronal)	-2.0
8026877	SLC5A5	solute carrier family 5 (sodium iodide symporter), member 5	-2.0
8166784	TSPAN7	tetraspanin 7	-2.0
7928882	C10orf116	chromosome 10 open reading frame 116	-2.0
8114612	CD14	CD14 molecule	-2.0
7943984	ZBTB16	zinc finger and BTB domain containing 16	-2.0
8005458	LGALS9C	lectin, galactoside-binding, soluble, 9C	-2.0
7962895	FKBP11	FK506 binding protein 11, 19 kDa	-2.1
7920642	MUC1	mucin 1, cell surface associated	-2.1
7968062	ATP12A	ATPase, H+	-2.1
8095697	CXCL1	chemokine (C-X-C motif) ligand 1 (melanoma growth stimulating activity, alpha)	-2.1

probeset_id	Symbol	Gene_Name	FC_T/N
8122202	MYB	v-myb myeloblastosis viral oncogene homolog (avian)	17.8
7912659	AGMAT	agmatine ureohydrolase (agmatinase)	-2.1
7919600	TRNAG34P	transfer RNA glycine 34 (anticodon CCC) pseudogene	-2.1
8030067	SULT2B1	sulfotransferase family, cytosolic, 2B, member 1	-2.1
8148476	DENND3	DENN	-2.1
7979241	BMP4	bone morphogenetic protein 4	-2.1
8013450	LGALS9	lectin, galactoside-binding, soluble, 9	-2.1
8144121	PTPRN2	protein tyrosine phosphatase, receptor type, N polypeptide 2	-2.1
8100994	CXCL2	chemokine (C-X-C motif) ligand 2	-2.1
8106999	C5orf27	chromosome 5 open reading frame 27	-2.1
7938299	RN5S330	RNA, 5S ribosomal 330	-2.1
8090823	SLCO2A1	solute carrier organic anion transporter family, member 2A1	-2.1
8098414	SPCS3	signal peptidase complex subunit 3 homolog (S. cerevisiae)	-2.1
8140463	FGL2	fibrinogen-like 2	-2.1
8101622	TECR	trans-2,3-enoyl-CoA reductase	-2.1
8034202	RAB3D	RAB3D, member RAS oncogene family	-2.1
8090343	KLF15	Kruppel-like factor 15	-2.1
8092978	MUC4	mucin 4, cell surface associated	-2.1
8006123	CPD	carboxypeptidase D	-2.1
8122334	CCRL1	chemokine (C-C motif) receptor-like 1	-2.1
8044080	SLC9A2	solute carrier family 9, subfamily A (NHE2, cation proton antiporter 2), member 2	-2.1
7983360	B2M	beta-2-microglobulin	-2.1
8115147	CD74	CD74 molecule, major histocompatibility complex, class II invariant chain	-2.1
7968015	TNFRSF19	tumor necrosis factor receptor superfamily, member 19	-2.1
8174513	CHRDL1	chordin-like 1	-2.1
7971015	SMAD9	SMAD family member 9	-2.1
7937079	BNIP3	BCL2	-2.1
7979473	DHRS7	dehydrogenase	-2.1
8013319	GRAP	GRB2-related adaptor protein	-2.1
7952022	AMICA1	adhesion molecule, interacts with CXADR antigen 1	-2.1
8059177	TUBA4A	tubulin, alpha 4a	-2.1
8041781	EPAS1	endothelial PAS domain protein 1	-2.1
7926786	APBB1IP	amyloid beta (A4) precursor protein-binding, family B, member 1 interacting protein	-2.1
7994769	CORO1A	coronin, actin binding protein, 1A	-2.1
8114215	PITX1	paired-like homeodomain 1	-2.1
8038785	VSIG10L	V-set and immunoglobulin domain containing 10 like	-2.1
8077899	PPARG	peroxisome proliferator-activated receptor gamma	-2.1
8000117	CRYM	crystallin, mu	-2.1
8156228	CTSL1	cathepsin L1	-2.1
8028600	NCCRP1	non-specific cytotoxic cell receptor protein 1 homolog (zebrafish)	-2.1
7932765	MPP7	membrane protein, palmitoylated 7 (MAGUK p55 subfamily member 7)	-2.1
8002020	TPPP3	tubulin polymerization-promoting protein family member 3	-2.1
8092177	NCEH1	neutral cholesterol ester hydrolase 1	-2.1

probeset_id	Symbol	Gene_Name	FC_T/N
8122202	MYB	v-myb myeloblastosis viral oncogene homolog (avian)	17.8
7906777	FCGR2B	Fc fragment of IgG, low affinity IIb, receptor (CD32)	-2.1
8096070	BMP3	bone morphogenetic protein 3	-2.1
7916364	LDLRAD1	low density lipoprotein receptor class A domain containing 1	-2.1
8153474	TSTA3	tissue specific transplantation antigen P35B	-2.1
8057599	TFPI	tissue factor pathway inhibitor (lipoprotein-associated coagulation inhibitor)	-2.1
8179481	HLA-DRA	major histocompatibility complex, class II, DR alpha	-2.1
7918457	KCNA3	potassium voltage-gated channel, shaker-related subfamily, member 3	-2.1
8042696	SPR	sepiapterin reductase (7,8-dihydrobiopterin:NADP+ oxidoreductase)	-2.1
8113214	GLRX	glutaredoxin (thioltransferase)	-2.1
8005097	HS3ST3B1	heparan sulfate (glucosamine) 3-O-sulfotransferase 3B1	-2.1
8153959	DOCK8	dedicator of cytokinesis 8	-2.1
7998233	TMEM8A	transmembrane protein 8A	-2.1
8092800	ATP13A4	ATPase type 13A4	-2.1
8096845	EGF	epidermal growth factor	-2.1
7905581	S100A1	S100 calcium binding protein A1	-2.1
7928046	TSPAN15	tetraspanin 15	-2.1
8115261	CCDC69	coiled-coil domain containing 69	-2.2
8152812	FAM84B	family with sequence similarity 84, member B	-2.2
7975268	ARG2	arginase, type II	-2.2
7952426	VSIG2	V-set and immunoglobulin domain containing 2	-2.2
7903893	CD53	CD53 molecule	-2.2
7958410	FICD	FIC domain containing	-2.2
8125537	HLA-DMA	major histocompatibility complex, class II, DM alpha	-2.2
8131550	SCIN	scinderin	-2.2
8056323	FIGN	fidgetin	-2.2
7973084	ANG	angiogenin, ribonuclease, RNase A family, 5	-2.2
8118345	CFB	complement factor B	-2.2
8157524	TLR4	toll-like receptor 4	-2.2
8126820	GPR110	G protein-coupled receptor 110	-2.2
7941148	TM7SF2	transmembrane 7 superfamily member 2	-2.2
8113369	SLCO4C1	solute carrier organic anion transporter family, member 4C1	-2.2
8097513	MGST2	microsomal glutathione S-transferase 2	-2.2
7962274	KIF21A	kinesin family member 21A	-2.2
7976783	DLK1	delta-like 1 homolog (Drosophila)	-2.2
7909877	MARC1	mitochondrial amidoxime reducing component 1	-2.2
7961365	MANSC1	MANSC domain containing 1	-2.2
7914342	FABP3	fatty acid binding protein 3, muscle and heart (mammary-derived growth inhibitor)	-2.2
8140166	RN5S233	RNA, 5S ribosomal 233	-2.2
8147661	SPAG1	sperm associated antigen 1	-2.2
8168470	COX7B	cytochrome c oxidase subunit VIIb	-2.2
8028332	KCNK6	potassium channel, subfamily K, member 6	-2.2
7929065	IFIT1	interferon-induced protein with tetratricopeptide repeats 1	-2.2

probeset_id	Symbol	Gene_Name	FC_T/N
8122202	MYB	v-myb myeloblastosis viral oncogene homolog (avian)	17.8
8122265	TNFAIP3	tumor necrosis factor, alpha-induced protein 3	-2.2
8152355	SYBU	syntabulin (syntaxin-interacting)	-2.2
7943413	BIRC3	baculoviral IAP repeat containing 3	-2.2
8037835	SLC1A5	solute carrier family 1 (neutral amino acid transporter), member 5	-2.2
8122196	RN5S218	RNA, 5S ribosomal 218	-2.2
7899023	LDLRAP1	low density lipoprotein receptor adaptor protein 1	-2.2
8041582	PKDCC	protein kinase domain containing, cytoplasmic homolog (mouse)	-2.2
7965357	GALNT4	UDP-N-acetyl-alpha-D-galactosamine:polypeptide N-acetylgalactosaminyltransferase 4 [illegible]	-2.2
8055872	CACNB4	calcium channel, voltage-dependent, beta 4 subunit	-2.2
7902518	GIPC2	GIPC PDZ domain containing family, member 2	-2.2
7944185	CD3G	CD3g molecule, gamma (CD3-TCR complex)	-2.2
8169174	RNF128	ring finger protein 128, E3 ubiquitin protein ligase	-2.2
8175177	MBNL3	muscleblind-like splicing regulator 3	-2.2
7917912	DPYD	dihydropyrimidine dehydrogenase	-2.2
8007043	RAPGEFL1	Rap guanine nucleotide exchange factor (GEF)-like 1	-2.2
7920082	RORC	RAR-related orphan receptor C	-2.2
7910377	RN5S19	RNA, 5S ribosomal 19	-2.2
8042310	SLC1A4	solute carrier family 1 (glutamate	-2.2
7995419	RN5S425	RNA, 5S ribosomal 425	-2.2
7956046	DGKA	diacylglycerol kinase, alpha 80kDa	-2.2
8066619	PLTP	phospholipid transfer protein	-2.3
8111677	LIFR	leukemia inhibitory factor receptor alpha	-2.3
8108631	VTRNA1-3	vault RNA 1-3	-2.3
7999253	PPL	periplakin	-2.3
8115623	ATP10B	ATPase, class V, type 10B	-2.3
7956878	IRAK3	interleukin-1 receptor-associated kinase 3	-2.3
8041225	EHD3	EH-domain containing 3	-2.3
8024062	CFD	complement factor D (adipsin)	-2.3
7930413	DUSP5	dual specificity phosphatase 5	-2.3
8056343	COBLL1	COBL-like 1	-2.3
7991034	HOMER2	homer homolog 2 (Drosophila)	-2.3
7908409	RGS2	regulator of G-protein signaling 2, 24kDa	-2.3
8152463	RN5S276	RNA, 5S ribosomal 276	-2.3
8101992	SLC39A8	solute carrier family 39 (zinc transporter), member 8	-2.3
8026861	B3GNT3	UDP-GlcNAc:betaGal beta-1,3-N-acetylglucosaminyltransferase 3	-2.3
8038747	KLK12	kallikrein-related peptidase 12	-2.3
8068383	CLIC6	chloride intracellular channel 6	-2.3
7936494	GFRA1	GDNF family receptor alpha 1	-2.3
7965040	PHLDA1	pleckstrin homology-like domain, family A, member 1	-2.3
8005638	ALDH3A2	aldehyde dehydrogenase 3 family, member A2	-2.3
8163618	TNFSF15	tumor necrosis factor (ligand) superfamily, member 15	-2.3
7939150	PRRG4	proline rich Gla (G-carboxyglutamic acid) 4 (transmembrane)	-2.3

probeset_id	Symbol	Gene_Name	FC_T/N
8122202	MYB	v-myb myeloblastosis viral oncogene homolog (avian)	17.8
8144880	SH2D4A	SH2 domain containing 4A	-2.3
7955348	GPD1	glycerol-3-phosphate dehydrogenase 1 (soluble)	-2.3
8156321	SYK	spleen tyrosine kinase	-2.3
7899160	CD52	CD52 molecule	-2.3
8016718	CHAD	chondroadherin	-2.3
7924450	DUSP10	dual specificity phosphatase 10	-2.3
7961075	CD69	CD69 molecule	-2.3
7934997	PPP1R3C	protein phosphatase 1, regulatory subunit 3C	-2.3
8070579	TFF1	trefoil factor 1	-2.3
8151310	EYA1	eyes absent homolog 1 (Drosophila)	-2.3
8143221	ATP6V0A4	ATPase, H+ transporting, lysosomal V0 subunit a4	-2.3
8009685	SLC9A3R1	solute carrier family 9, subfamily A (NHE3, cation proton antiporter 3), member 3 regulator 1	-2.3
8084710	ADIPOQ	adiponectin, C1Q and collagen domain containing	-2.3
8011671	GGT6	gamma-glutamyltransferase 6	-2.3
8149811	NKX3-1	NK3 homeobox 1	-2.3
7931832	LOC100653286	aldo-keto reductase family 1 member C2-like	-2.3
8146906	RN5S271	RNA, 5S ribosomal 271	-2.3
7965964	SLC41A2	solute carrier family 41, member 2	-2.3
8000346	ERN2	endoplasmic reticulum to nucleus signaling 2	-2.3
8058627	ERBB4	v-erb-a erythroblastic leukemia viral oncogene homolog 4 (avian)	-2.3
8156761	NANS	N-acetylneuraminic acid synthase	-2.3
8155849	ANXA1	annexin A1	-2.3
8161945	RASEF	RAS and EF-hand domain containing	-2.3
7912706	EPHA2	EPH receptor A2	-2.3
8173745	CYSLTR1	cysteinyl leukotriene receptor 1	-2.3
8028652	ZFP36	zinc finger protein 36, C3H type, homolog (mouse)	-2.4
8072170	KREMEN1	kringle containing transmembrane protein 1	-2.4
8082797	TF	transferrin	-2.4
8139712	VOPP1	vesicular, overexpressed in cancer, prosurvival protein 1	-2.4
8009301	PRKCA	protein kinase C, alpha	-2.4
8152606	SNTB1	syntrophin, beta 1 (dystrophin-associated protein A1, 59kDa, basic component 1)	-2.4
8084717	ST6GAL1	ST6 beta-galactosamide alpha-2,6-sialyltranferase 1	-2.4
8174361	TSC22D3	TSC22 domain family, member 3	-2.4
7907702	SOAT1	sterol O-acyltransferase 1	-2.4
7971690	RN5S29	RNA, 5S ribosomal 29	-2.4
8161892	GNA14	guanine nucleotide binding protein (G protein), alpha 14	-2.4
8047272	SPATS2L	spermatogenesis associated, serine-rich 2-like	-2.4
7929816	SCD	stearoyl-CoA desaturase (delta-9-desaturase)	-2.4
8011516	ATP2A3	ATPase, Ca++ transporting, ubiquitous	-2.4
7968789	RGCC	regulator of cell cycle	-2.4
8161568	LOC642424	ig kappa chain V-I region Walker-like	-2.4
8059580	DNER	delta	-2.4

probeset_id	Symbol	Gene_Name	FC_T/N
8122202	MYB	v-myb myeloblastosis viral oncogene homolog (avian)	17.8
8108447	CXXC5	CXXC finger protein 5	-2.4
8104447	RN5S177	RNA, 5S ribosomal 177	-2.4
8013384	ALDH3A1	aldehyde dehydrogenase 3 family, member A1	-2.4
7958913	OAS2	2'-5'-oligoadenylate synthetase 2, 69	-2.4
7994265	RN5S405	RNA, 5S ribosomal 405	-2.5
8119016	MAPK13	mitogen-activated protein kinase 13	-2.5
8081288	TMEM45A	transmembrane protein 45A	-2.5
7922174	F5	coagulation factor V (proaccelerin, labile factor)	-2.5
7975076	HSPA2	heat shock 70kDa protein 2	-2.5
8108912	SH3RF2	SH3 domain containing ring finger 2	-2.5
7966259	GLTP	glycolipid transfer protein	-2.5
8114572	HBEGF	heparin-binding EGF-like growth factor	-2.5
8003156	RN5S433	RNA, 5S ribosomal 433	-2.5
7942957	PRSS23	protease, serine, 23	-2.5
7997662	KIAA0513	KIAA0513	-2.5
7995362	GPT2	glutamic pyruvate transaminase (alanine aminotransferase) 2	-2.5
8121838	TPD52L1	tumor protein D52-like 1	-2.5
8096602	DAPP1	dual adaptor of phosphotyrosine and 3-phosphoinositides	-2.5
8016390	COPZ2	coatomer protein complex, subunit zeta 2	-2.5
7903227	PALMD	palmdelphin	-2.5
8131600	TSPAN13	tetraspanin 13	-2.5
8001547	PLLP	plasmolipin	-2.5
7949971	CPT1A	carnitine palmitoyltransferase 1A (liver)	-2.5
7981720	IGHV3-35	immunoglobulin heavy variable 3-35 (non-functional)	-2.5
8171624	GPR64	G protein-coupled receptor 64	-2.5
8059642	SLC16A14	solute carrier family 16, member 14 (monocarboxylic acid transporter 14)	-2.5
8125919	FKBP5	FK506 binding protein 5	-2.5
8041542	GALM	galactose mutarotase (aldose 1-epimerase)	-2.5
7967318	HCAR2	hydroxycarboxylic acid receptor 2	-2.5
8008588	HLF	hepatic leukemia factor	-2.5
8134834	AGFG2	ArfGAP with FG repeats 2	-2.6
8040249	ATP6V1C2	ATPase, H+ transporting, lysosomal 42kDa, V1 subunit C2	-2.6
8155930	GCNT1	glucosaminyl (N-acetyl) transferase 1, core 2	-2.6
8017867	FAM20A	family with sequence similarity 20, member A	-2.6
8101762	SNCA	synuclein, alpha (non A4 component of amyloid precursor)	-2.6
8170119	FHL1	four and a half LIM domains 1	-2.6
7907260	FMO6P	flavin containing monooxygenase 6 pseudogene	-2.6
8021301	RAB27B	RAB27B, member RAS oncogene family	-2.6
8047300	AOX1	aldehyde oxidase 1	-2.6
8078971	ENTPD3	ectonucleoside triphosphate diphosphohydrolase 3	-2.6
8078933	MYRIP	myosin VIIA and Rab interacting protein	-2.6
8007493	ARL4D	ADP-ribosylation factor-like 4D	-2.6

probeset_id	Symbol	Gene_Name	FC_T/N
8122202	MYB	v-myb myeloblastosis viral oncogene homolog (avian)	17.8
8023497	ATP8B1	ATPase, aminophospholipid transporter, class I, type 8B, member 1	-2.6
8048717	SGPP2	sphingosine-1-phosphate phosphatase 2	-2.6
8073680	TRNAU2	transfer RNA selenocysteine 2 (anticodon UCA)	-2.6
8029136	CD79A	CD79a molecule, immunoglobulin-associated alpha	-2.6
8081171	CRYBG3	beta-gamma crystallin domain containing 3	-2.6
8052355	EFEMP1	EGF containing fibulin-like extracellular matrix protein 1	-2.6
7935337	PIK3AP1	phosphoinositide-3-kinase adaptor protein 1	-2.7
8143144	PTN	pleiotrophin	-2.7
7981068	SERPINA1	serpin peptidase inhibitor, clade A (alpha-1 antiproteinase, antitrypsin), member 1	-2.7
8108627	VTRNA1-1	vault RNA 1-1	-2.7
7913385	RAP1GAP	RAP1 GTPase activating protein	-2.7
8070567	TFF3	trefoil factor 3 (intestinal)	-2.7
7913667	GALE	UDP-galactose-4-epimerase	-2.7
8033987	ICAM3	intercellular adhesion molecule 3	-2.7
7936463	ABLIM1	actin binding LIM protein 1	-2.7
7974835	PRKCH	protein kinase C, eta	-2.7
8133721	HSPB1	heat shock 27kDa protein 1	-2.7
7970441	GJB2	gap junction protein, beta 2, 26kDa	-2.7
7901256	CYP4B1	cytochrome P450, family 4, subfamily B, polypeptide 1	-2.7
7950906	CTSC	cathepsin C	-2.7
8021528	TNFRSF11A	tumor necrosis factor receptor superfamily, member 11a, NFKB activator	-2.7
8078386	GPD1L	glycerol-3-phosphate dehydrogenase 1-like	-2.7
8014316	CCL5	chemokine (C-C motif) ligand 5	-2.7
8113220	ELL2	elongation factor, RNA polymerase II, 2	-2.7
8043484	IGKV1OR2-118	immunoglobulin kappa variable 1	-2.7
8002882	CHST6	carbohydrate (N-acetylglucosamine 6-O) sulfotransferase 6	-2.7
8103951	ACSL1	acyl-CoA synthetase long-chain family member 1	-2.7
8055980	CYTIP	cytohesin 1 interacting protein	-2.7
8121257	PRDM1	PR domain containing 1, with ZNF domain	-2.7
7938629	PDE3B	phosphodiesterase 3B, cGMP-inhibited	-2.7
8116439	SCGB3A1	secretoglobin, family 3A, member 1	-2.7
7958884	OAS1	2'-5'-oligoadenylate synthetase 1, 40	-2.7
8174277	RN5S511	RNA, 5S ribosomal 511	-2.7
8146934	LY96	lymphocyte antigen 96	-2.7
7991762	HBA1	hemoglobin, alpha 1	-2.7
7956826	TBC1D30	TBC1 domain family, member 30	-2.8
7922976	PTGS2	prostaglandin-endoperoxide synthase 2 (prostaglandin G	-2.8
8103769	HPGD	hydroxyprostaglandin dehydrogenase 15-(NAD)	-2.8
8038633	KLK1	kallikrein 1	-2.8
8134452	BHLHA15	basic helix-loop-helix family, member a15	-2.8
8101828	TSPAN5	tetraspanin 5	-2.8
7932160	FAM107B	family with sequence similarity 107, member B	-2.8

probeset_id	Symbol	Gene_Name	FC_T/N
8122202	MYB	v-myb myeloblastosis viral oncogene homolog (avian)	17.8
8163257	LPAR1	lysophosphatidic acid receptor 1	-2.8
8056545	STK39	serine threonine kinase 39	-2.8
7961320	PRB1	proline-rich protein BstNI subfamily 1	-2.8
7909214	RASSF5	Ras association (RalGDS	-2.8
8153002	NDRG1	N-myc downstream regulated 1	-2.8
7996563	HSD11B2	hydroxysteroid (11-beta) dehydrogenase 2	-2.8
8098611	TLR3	toll-like receptor 3	-2.8
7928944	PAPSS2	3'-phosphoadenosine 5'-phosphosulfate synthase 2	-2.9
8091537	IGSF10	immunoglobulin superfamily, member 10	-2.9
7983512	SQRDL	sulfide quinone reductase-like (yeast)	-2.9
7928543	RN5S321	RNA, 5S ribosomal 321	-2.9
8068583	KCNJ15	potassium inwardly-rectifying channel, subfamily J, member 15	-2.9
8161174	GNE	glucosamine (UDP-N-acetyl)-2-epimerase	-2.9
8032392	MKNK2	MAP kinase interacting serine	-2.9
8113512	EPB41L4A	erythrocyte membrane protein band 4.1 like 4A	-2.9
8163002	KLF4	Kruppel-like factor 4 (gut)	-2.9
7988990	WDR72	WD repeat domain 72	-2.9
8126891	CRISP2	cysteine-rich secretory protein 2	-2.9
7934898	ANKRD22	ankyrin repeat domain 22	-2.9
7984001	GCNT3	glucosaminyl (N-acetyl) transferase 3, mucin type	-2.9
7977933	SLC7A8	solute carrier family 7 (amino acid transporter light chain, L system), member 8	-2.9
8101904	ADH7	alcohol dehydrogenase 7 (class IV), mu or sigma polypeptide	-3.0
8090314	ALDH1L1	aldehyde dehydrogenase 1 family, member L1	-3.0
8071658	IGLV7-46	immunoglobulin lambda variable 7-46 (gene	-3.0
8115831	DUSP1	dual specificity phosphatase 1	-3.0
7919800	CTSS	cathepsin S	-3.0
8033043	FUT6	fucosyltransferase 6 (alpha (1,3) fucosyltransferase)	-3.0
7922130	DPT	dermatopontin	-3.0
8120961	MRAP2	melanocortin 2 receptor accessory protein 2	-3.0
8049487	MLPH	melanophilin	-3.0
7919055	HMGCS2	3-hydroxy-3-methylglutaryl-CoA synthase 2 (mitochondrial)	-3.0
8005879	SLC13A2	solute carrier family 13 (sodium-dependent dicarboxylate transporter), member 2	-3.0
8146115	C8orf4	chromosome 8 open reading frame 4	-3.0
8129254	MAN1A1	mannosidase, alpha, class 1A, member 1	-3.0
7960362	LOC100128816	ACAH3104	-3.0
8030094	FUT2	fucosyltransferase 2 (secretor status included)	-3.0
7915910	PDZK1IP1	PDZK1 interacting protein 1	-3.0
8052940	PAIP2B	poly(A) binding protein interacting protein 2B	-3.1
8144786	SLC7A2	solute carrier family 7 (cationic amino acid transporter, y+ system), member 2	-3.1
8101881	ADH1B	alcohol dehydrogenase 1B (class I), beta polypeptide	-3.1
7917276	LPAR3	lysophosphatidic acid receptor 3	-3.1
7958784	ALDH2	aldehyde dehydrogenase 2 family (mitochondrial)	-3.1

probeset_id	Symbol	Gene_Name	FC_T/N
8122202	MYB	v-myb myeloblastosis viral oncogene homolog (avian)	17.8
8127854	ME1	malic enzyme 1, NADP(+)-dependent, cytosolic	-3.1
8089145	ABI3BP	ABI family, member 3 (NESH) binding protein	-3.1
7916432	DHCR24	24-dehydrocholesterol reductase	-3.1
8103226	TMEM154	transmembrane protein 154	-3.1
8141094	PDK4	pyruvate dehydrogenase kinase, isozyme 4	-3.2
8045882	DAPL1	death associated protein-like 1	-3.2
7981722	IGHV3-38	immunoglobulin heavy variable 3-38 (non-functional)	-3.2
7955297	AQP5	aquaporin 5	-3.2
8150889	SDR16C5	short chain dehydrogenase	-3.2
7946579	LYVE1	lymphatic vessel endothelial hyaluronan receptor 1	-3.2
8001457	CES1	carboxylesterase 1	-3.2
8157264	SLC31A2	solute carrier family 31 (copper transporters), member 2	-3.2
7922598	ANGPTL1	angiopoietin-like 1	-3.2
8165453	LRRC26	leucine rich repeat containing 26	-3.2
8147132	CA2	carbonic anhydrase II	-3.2
8053713	IGKV2-10	immunoglobulin kappa variable 2-10 (pseudogene)	-3.2
7979505	SIX1	SIX homeobox 1	-3.2
8002303	NQO1	NAD(P)H dehydrogenase, quinone 1	-3.2
8160889	CCL21	chemokine (C-C motif) ligand 21	-3.2
7920297	S100A14	S100 calcium binding protein A14	-3.3
8046099	NOSTRIN	nitric oxide synthase trafficker	-3.3
7981737	IGHV3-72	immunoglobulin heavy variable 3-72	-3.3
8043433	IGKC	immunoglobulin kappa constant	-3.3
7913593	TCEA3	transcription elongation factor A (SII), 3	-3.3
8101429	PLAC8	placenta-specific 8	-3.3
7979179	ERO1L	ERO1-like (S. cerevisiae)	-3.3
8092726	CLDN1	claudin 1	-3.3
7988414	GATM	glycine amidinotransferase (L-arginine:glycine amidinotransferase)	-3.3
8123598	SERPINB1	serpin peptidase inhibitor, clade B (ovalbumin), member 1	-3.4
7963313	GALNT6	UDP-N-acetyl-alpha-D-galactosamine:polypeptide N-acetylgalactosaminyltransferase 6 (GalNAc-T6)	-3.4
8043474	IGKV1D-42	immunoglobulin kappa variable 1D-42 (non-functional)	-3.4
8081710	SIDT1	SID1 transmembrane family, member 1	-3.4
7906613	SLAMF7	SLAM family member 7	-3.4
7979658	GPX2	glutathione peroxidase 2 (gastrointestinal)	-3.4
8043360	IGKV3-7	immunoglobulin kappa variable 3-7 (non-functional)	-3.5
8067125	BCAS1	breast carcinoma amplified sequence 1	-3.5
8142585	CADPS2	Ca++-dependent secretion activator 2	-3.5
8106448	PDE8B	phosphodiesterase 8B	-3.5
8043459	IGKV1D-16	immunoglobulin kappa variable 1D-16	-3.5
7946033	HBB	hemoglobin, beta	-3.5
8135544	FOXP2	forkhead box P2	-3.5
8163181	C9orf152	chromosome 9 open reading frame 152	-3.5

probeset_id	Symbol	Gene_Name	FC_T/N
8122202	MYB	v-myb myeloblastosis viral oncogene homolog (avian)	17.8
8161884	PRUNE2	prune homolog 2 (Drosophila)	-3.5
7925452	GREM2	gremlin 2	-3.5
7907160	ATP1B1	ATPase, Na+	-3.5
7938687	NUCB2	nucleobindin 2	-3.5
7981732	IGHV4-61	immunoglobulin heavy variable 4-61	-3.5
8075182	XBP1	X-box binding protein 1	-3.6
7951686	IL18	interleukin 18 (interferon-gamma-inducing factor)	-3.6
7944931	SLC37A2	solute carrier family 37 (glycerol-3-phosphate transporter), member 2	-3.6
8118069	MUC21	mucin 21, cell surface associated	-3.6
8038735	KLK11	kallikrein-related peptidase 11	-3.6
8088180	WNT5A	wingless-type MMTV integration site family, member 5A	-3.6
7960365	EFCAB4B	EF-hand calcium binding domain 4B	-3.6
8043443	IGKV2-24	immunoglobulin kappa variable 2-24	-3.6
7944164	TMPRSS4	transmembrane protease, serine 4	-3.6
8163908	GGTA1P	glycoprotein, alpha-galactosyltransferase 1 pseudogene	-3.7
8140840	STEAP4	STEAP family member 4	-3.7
8029086	CEACAM5	carcinoembryonic antigen-related cell adhesion molecule 5	-3.7
8103877	CLDN22	claudin 22	-3.7
7922846	FAM129A	family with sequence similarity 129, member A	-3.7
8043446	IGKV6-21	immunoglobulin kappa variable 6-21 (non-functional)	-3.7
8122071	ENPP3	ectonucleotide pyrophosphatase	-3.7
7981730	IGLJ3	immunoglobulin lambda joining 3	-3.7
7991283	RHCG	Rh family, C glycoprotein	-3.8
8033054	FUT3	fucosyltransferase 3 (galactoside 3(4)-L-fucosyltransferase, Lewis blood group)	-3.8
8156770	GALNT12	UDP-N-acetyl-alpha-D-galactosamine:polypeptide N-acetylgalactosaminyltransferase 12 [illegible]	-3.8
8125843	SPDEF	SAM pointed domain containing ets transcription factor	-3.8
8018774	ST6GALNAC1	ST6 (alpha-N-acetyl-neuraminyl-2,3-beta-galactosyl-1,3)-N-acetylgalactosaminide alpha-2,6- [illegible]	-3.8
7944023	NXPE2	neurexophilin and PC-esterase domain family, member 2	-3.8
8173444	IL2RG	interleukin 2 receptor, gamma	-3.8
7973974	PAX9	paired box 9	-3.8
8043470	IGKV3D-11	immunoglobulin kappa variable 3D-11	-3.9
8021453	SEC11C	SEC11 homolog C (S. cerevisiae)	-3.9
8162502	FBP1	fructose-1,6-bisphosphatase 1	-3.9
8106354	IQGAP2	IQ motif containing GTPase activating protein 2	-3.9
8128123	RRAGD	Ras-related GTP binding D	-3.9
8096459	RN5S164	RNA, 5S ribosomal 164	-3.9
8172204	MAOB	monoamine oxidase B	-4.0
7959102	HSPB8	heat shock 22kDa protein 8	-4.0
7965979	ALDH1L2	aldehyde dehydrogenase 1 family, member L2	-4.0
8043449	IGK@	immunoglobulin kappa locus	-4.0
8120833	SH3BGRL2	SH3 domain binding glutamic acid-rich protein like 2	-4.0
8043465	IGKV1D-13	immunoglobulin kappa variable 1D-13	-4.0

probeset_id	Symbol	Gene_Name	FC_T/N
8122202	MYB	v-myb myeloblastosis viral oncogene homolog (avian)	17.8
7902738	CLCA4	chloride channel accessory 4	-4.0
7993638	TMC5	transmembrane channel-like 5	-4.1
8078227	KAT2B	K(lysine) acetyltransferase 2B	-4.1
8133876	CD36	CD36 molecule (thrombospondin receptor)	-4.1
7961252	PRR4	proline rich 4 (lacrimal)	-4.1
8149927	CLU	clusterin	-4.1
7983239	CKMT1A	creatine kinase, mitochondrial 1A	-4.1
8084165	SOX2	SRY (sex determining region Y)-box 2	-4.1
7987385	MEIS2	Meis homeobox 2	-4.2
7904361	FAM46C	family with sequence similarity 46, member C	-4.2
8136709	LOC93432	maltase-glucoamylase (alpha-glucosidase) pseudogene	-4.2
8045835	GALNT5	UDP-N-acetyl-alpha-D-galactosamine:polypeptide N-acetylgalactosaminyltransferase 5 [illegible]	-4.3
7939642	CREB3L1	cAMP responsive element binding protein 3-like 1	-4.3
7960529	SCNN1A	sodium channel, non-voltage-gated 1 alpha subunit	-4.3
7923850	SLC26A9	solute carrier family 26, member 9	-4.3
8095451	C4orf40	chromosome 4 open reading frame 40	-4.3
8037197	CXCL17	chemokine (C-X-C motif) ligand 17	-4.4
7981740	IGHV3-74	immunoglobulin heavy variable 3-74	-4.4
8066493	SLPI	secretory leukocyte peptidase inhibitor	-4.5
8123246	SLC22A3	solute carrier family 22 (extraneuronal monoamine transporter), member 3	-4.6
8090433	MGLL	monoglyceride lipase	-4.7
7947156	MUC15	mucin 15, cell surface associated	-4.7
8043476	IGKV1D-43	immunoglobulin kappa variable 1D-43	-4.7
7981718	IGHV3-33	immunoglobulin heavy variable 3-33	-4.7
8095488	SMR3A	submaxillary gland androgen regulated protein 3A	-4.8
7964722	WIF1	WNT inhibitory factor 1	-4.8
7966749	TESC	tescalcin	-4.9
8043431	IGKV1-33	immunoglobulin kappa variable 1-33	-4.9
8061847	BPIFA2	BPI fold containing family A, member 2	-5.0
8158167	LCN2	lipocalin 2	-5.0
8138289	ETV1	ets variant 1	-5.0
7945169	TMEM45B	transmembrane protein 45B	-5.1
7981728	IGHV3-52	immunoglobulin heavy variable 3-52 (pseudogene)	-5.1
8043436	IGKV2D-29	immunoglobulin kappa variable 2D-29	-5.2
8082673	ACPP	acid phosphatase, prostate	-5.2
8148059	DEPTOR	DEP domain containing MTOR-interacting protein	-5.3
8095751	PARM1	prostate androgen-regulated mucin-like protein 1	-5.3
8095467	FDCSP	follicular dendritic cell secreted protein	-5.5
8043468	IGKV1-12	immunoglobulin kappa variable 1-12	-5.7
8029098	CEACAM6	carcinoembryonic antigen-related cell adhesion molecule 6 (non-specific cross reacting [illegible]	-5.8
8088425	FAM3D	family with sequence similarity 3, member D	-6.2
7964927	TSPAN8	tetraspanin 8	-6.2

probeset_id	Symbol	Gene_Name	FC_T/N
8122202	MYB	v-myb myeloblastosis viral oncogene homolog (avian)	17.8
8101893	ADH1C	alcohol dehydrogenase 1C (class I), gamma polypeptide	-6.3
8095380	TMPRSS11E	transmembrane protease, serine 11E	-6.4
8095435	HTN1	histatin 1	-6.5
8008736	LPO	lactoperoxidase	-6.6
8149097	DEFB1	defensin, beta 1	-6.8
8043441	IGKV1D-27	immunoglobulin kappa variable 1D-27 (pseudogene)	-6.8
7989501	CA12	carbonic anhydrase XII	-6.9
7992732	ZG16B	zymogen granule protein 16 homolog B (rat)	-6.9
8151532	FABP4	fatty acid binding protein 4, adipocyte	-7.1
7901175	TSPAN1	tetraspanin 1	-7.2
8160670	AQP3	aquaporin 3 (Gill blood group)	-7.3
8056222	DPP4	dipeptidyl-peptidase 4	-7.5
8061780	BPIFB2	BPI fold containing family B, member 2	-7.6
7948444	TCN1	transcobalamin I (vitamin B12 binding protein, R binder family)	-7.6
8161755	ALDH1A1	aldehyde dehydrogenase 1 family, member A1	-7.7
8173869	POF1B	premature ovarian failure, 1B	-8.6
8095491	SMR3B	submaxillary gland androgen regulated protein 3B	-9.0
8095456	ODAM	odontogenic, ameloblast asssociated	-9.1
8138381	AGR2	anterior gradient 2 homolog (Xenopus laevis)	-9.3
8100827	IGJ	immunoglobulin J polypeptide, linker protein for immunoglobulin alpha and mu polypeptides	-9.6
8068684	FAM3B	family with sequence similarity 3, member B	-10.1
8061894	BPIFB1	BPI fold containing family B, member 1	-10.8
8095504	MUC7	mucin 7, secreted	-10.9
7957023	LYZ	lysozyme	-11.2
7923929	PIGR	polymeric immunoglobulin receptor	-12.8
7931108	DMBT1	deleted in malignant brain tumors 1	-13.2
8095422	STATH	statherin	-17.5
8136839	PIP	prolactin-induced protein	-20.7
8126905	CRISP3	cysteine-rich secretory protein 3	-22.4

Supplementary Table 2: Siginificantly expressed miRNAs in adenoid cystic cancers compared to mathced normal tissues. Paired t-test, FDR=0.05; Baseline filter: max{tumor, normal} >=800; Variation filter: |fold change|>=2.0.

Probe_id	avg_Tumor Intensity	avg_Normal Intensity	Fold Change (T/N)
hsa-miR-375_st	141.5	2246.3	-15.9
hsa-miR-31_st	166.6	1595.7	-9.6
hsa-miR-150_st	157.4	932.0	-5.9
hsa-miR-152_st	360.8	1091.2	-3.0
hsa-miR-214_st	1110.2	2843.3	-2.6
hsa-miR-29a_st	703.1	1775.7	-2.5
hsa-miR-126_st	1107.0	2601.3	-2.3
hsa-miR-140-3p_st	1495.8	3500.5	-2.3
hsa-miR-199b-3p_st	818.9	1736.1	-2.1
hsa-miR-923_st	2063.4	4337.1	-2.1
hsa-miR-199a-3p_st	851.2	1730.1	-2.0
hsa-miR-652_st	857.6	367.9	2.3
hsa-miR-130a_st	1506.2	618.4	2.4
hsa-miR-93_st	6382.9	2530.1	2.5
hsa-miR-182_st	2689.9	1048.5	2.6
hsa-miR-181a_st	5502.4	1984.0	2.8
hsa-miR-106b_st	3972.5	1422.5	2.8
hsa-miR-146a_st	2791.2	979.5	2.8
hsa-miR-25_st	1687.7	533.7	3.2
hsa-miR-744_st	820.3	250.3	3.3
hsa-miR-181b_st	2799.3	597.0	4.7
hsa-miR-455-3p_st	7361.8	549.1	13.4
